# The sediment transport mechanics driving lateral accretion in muddy meanders

**DOI:** 10.1073/pnas.2506462122

**Published:** 2025-07-01

**Authors:** Runze Miao, Alan D. Howard, William E. Dietrich

**Affiliations:** ^a^Department of Earth and Planetary Science, University of California, Berkeley, CA 94720; ^b^Department of Environmental Sciences, University of Virginia, Charlottesville, VA 22904; ^c^Planetary Science Institute, Tucson, AZ 85719

**Keywords:** muddy meanders, suspended sediment, lateral accretion, basal concentration, mud supply

## Abstract

The discovery of paleo-meanders on Mars and debates about the occurrence of meandering on prevegetation early Earth have stimulated field studies of channel morphology and deposit stratigraphy in arid environments. They show that mud can provide the cohesive strength to retard outer bank erosion, enabling inner bank lateral accretion (including mud deposition) to keep pace and thus sustain meandering. The process by which mud is deposited in the lateral accretion deposits, however, is poorly known. Building on previous fieldwork in the muddy meandering Quinn River, we use Delft3D and the Partheniades–Krone model for cohesive sediment transport to predict erosion and deposition patterns in a 2,300 m long reach. While flocculation of mud increases the settling velocity and hence the likelihood of deposition, the standard metric for distinguishing washload from suspended load, (i.e. settling velocity to shear velocity ratio), does not indicate relative deposition rate. Mud deposition is driven by the product of settling velocity times basal concentration: $w_{s}c_{b}$. Relatively low $w_{s}$ but high $c_{b}$ can induce deposition. We show that with sufficiently high basal concentrations, significant net lateral accretion deposition occurs in areas where elevated flocculation settling velocities are >100 times lower than the local shear velocity. Observed grain size distributions of lateral accretion deposits are predicted. Just by introducing a period of elevated mud load with no change in discharge creates a distinct muddy interbed. Our model points to multiple ways lateral accretion deposits can vary sedimentologically, including creating mud-sand couplets, and lead to single thread, active meandering channels in muddy rivers.

## Significance

Meandering rivers, common on Earth, are typically bordered by vegetation which both provides strength to outer banks and induces deposition along inner ones. River meander deposits are rare in early Earth geologic record before the rise of vegetation, but on Mars ancient meander tracks have been found. Mud can provide strength to the outer bank, but fine particles settle slowly making deposition less likely. Our model shows that despite the slow settling velocities, with elevated mud concentration, deposition will occur and sustain muddy meandering. Just briefly increasing the mud load for a constant discharge creates a distinct muddy layer in the inner bank deposit, thus explaining a common characteristic of meandering rivers found in the geologic record.

In meandering rivers, bank strength retards outer bank erosion rates, enabling inner bank deposition to keep pace with bank erosion, thus preserving channel form as rivers sweep back and forth building and eroding floodplain materials (e.g. ref. 1). Vegetation commonly provides this strength, and upon bank collapse that strength is lost, only to be gained by growth on freshly deposited inner bank deposits (that will ultimately become outer bank materials during shifting meandering dynamics). Experiments and modeling have advanced understanding of these processes as influenced by vegetation (e.g. refs. 2–4). The discovery, then, of traces of paleo-meandering rivers on Mars (e.g. refs. 5–9) was a surprise and consequently mud (sediment sizes less than 62.5 microns) was proposed as an alternative control on bank strength (e.g. refs. 5 and 7). Similarly, it has been argued that before the evolution of deep-rooted vegetation (pre-Silurian) on Earth, meandering channels were much less common and but may have developed where channel bank strength was provided by mud (e.g. refs. 10 and 11). Mud production may have been due to increased weathering effects of early plant life and deposition facilitated by organic compounds inducing flocculation (12). Questions about Mars and early-Earth river mechanics have led to field studies to characterize rivers in arid landscapes free of the influence of vegetation, and these studies have highlighted the role of mud in contributing to bank strength (e.g. refs. 7 and 13).

While mud strengthening of banks is well documented, it is less clear what controls mud deposition along the inner bank where lateral accretion deposits develop and track outer bank retreat. A now standard stratigraphic model for sand bedded river meanders consists of lateral accretion deposits composed of overlapping inclined beds with periodic interbeds of varying thickness of mud-rich layers, recording the lateral advance of point bars (e.g. ref. 14). Thomas et al. (15) introduced the term inclined heterolithic stratification to describe this layering. They proposed that the alternating layers of sand and mud may record elevated discharge events (depositing sand) followed by significantly reduced flow allowing mud to settle out and form a mud drape, or they can be a signature of tidally influenced mud deposition. Debate remains interpreting such layering in ancient deposits as being due to fluvial discharge variation or tidal dominance (e.g. ref. 16). Thin muddy beds (mud drapes) in sandy meandering river deposits retard fluid movement and the transfer of $\;{\text{CO}}_{2}$ and oil. Consequently, there is pragmatic interest in modeling their occurrence in deposits. For example, Hu et al. (17) introduced a rules-based stochastic model that adds mud drapes to modeled lateral accretion deposits in meandering rivers to model point bar reservoirs.

Sediment transport mechanics in muddy rivers have received little field or theoretical investigation. Such rivers are not discussed by Seminara et al. (1), and Finotello et al. (18) only mention the role mud can play in bank strength. Notably, however, Page et al. (19) showed that Australian muddy meandering rivers commonly lack well-formed point bars, but do deposit inclined lateral accretion deposits that can be mud rich. They referred to such deposits as oblique accretion.

The settling velocities of fine silt and clay are of such slow rates relative to the turbulence intensities of river flows, that significant deposition in active flows supporting sand transport, however, is generally not expected. Hence, mud is generally assumed to be washload, contributing little to net bed deposition (e.g. refs. 20 and 21). Studies have emphasized, however, that mud in rivers can be strongly flocculated, raising settling velocities by orders of magnitude and thus should lead to mud deposition (e.g. refs. 7 and 22). Sizeable flocs can also form and travel as bedload (23, 24).

Although much attention has been given to mud flocculation, increased settling velocities, and interaction with the bed, there has been no mechanistic numerical model applied to predict the patterns of mud deposition in river meanders. Here, we build upon prior investigations of the Quinn River, Nevada (7, 25), a muddy meandering river free of significant influence of vegetation, to apply a widely used morphodynamic model, Delft3D, to explore what controls mud deposition in lateral accretion deposits. We use the Delft3D application of a widely used cohesive sediment erosion and deposition model to predict observed patterns of mud deposition. Importantly, we show that it is not just settling velocity that matters. Sufficiently high sediment concentration can cause significant mud deposition even where settling velocities are far below shear velocities. This explains how mud is deposited in lateral accretion deposits in active currents in rivers, not just in slack water, and reveals how just varying the mud supply to rivers can generate distinct mud interbeds.

## Field Site

**Fig. 1.**
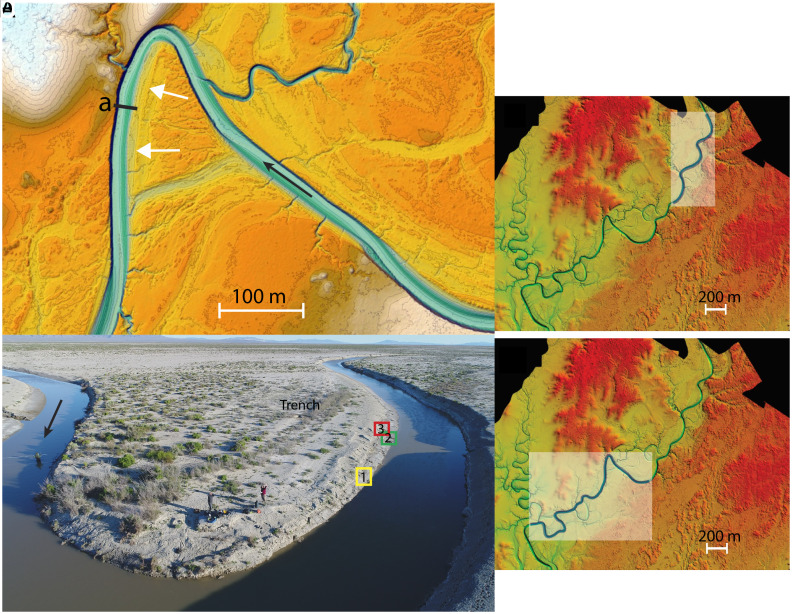
Quinn River study site. (*A*) Howard’s Bend colorized elevation with 20 cm contour lines. Brown to light tone topography is elevated topography underlain by Lake Lahontan sediment. White arrows point to lateral accretion deposits. Chute channel has developed across the bend. Section (a) locates cross-section shown in Fig. 3. The black arrow indicates the flow direction. (*B*) Drone view of Howard’s Bend during low flow field work; flow *Left* to *Right*. Number identifies locations of lateral accretion deposit and trench across a scroll bar. The Delft3D was run for two reaches shown in (*C* and *D*). Reach (*C*) model run compared flow and suspended sediment transport at a section with field measurement. Reach (*D*) includes the large Howard’s bend.

The Quinn River runs southward across the Basin and Range topography of western Nevada, the valley floor of which was flooded by Pleistocene Lake Lahontan (26), which left extensive mud deposits (*SI Appendix*, Fig. S1). In the lower reaches the meandering river has cut down and laterally, generating a floodplain where channel bends are variably bordered by earlier fluvial deposits and exposed Lake Lahontan sediments (Fig. 1 and *SI Appendix*, Figs. S2 and S3). The Quinn river terminates in the Black Rock Desert playa. Matsubara et al. (7, 25) selected a study reach (with an upslope drainage area of 12,154 $\;{\text{km}}^{2}$) (*SI Appendix*, Fig. S4) with traces of meander cutoffs (some occurring in recent decades, with the most recent in 2020), and abundant scroll bar features (Fig. 1 and *SI Appendix*, Figs. S2 and S3). Vegetation is sparse and roots are few and have no visible influence on bank strength (*SI Appendix*, Fig. S5). Instead, all samples of bed, alluvial banks, lateral accretion deposits, and floodplain deposits contain mud and fine sand, and outer banks of bends form vertical walls resulting from significant cohesion (7, 25). The mud is derived from the entrenched Lake Lahontan sediments and the fine sand is the only coarse sediment that arrives from upslope, as the flat lying bordering Lahontan deposit mostly prevents coarse sediment delivery from adjacent uplands. Point bars are essentially absent, but lateral accretion deposits are composed of inclined muddy sands with periodic distinct centimeters thick interbeds sandy muds (*SI Appendix*, Fig. S6). Floodplain overbank flows spill sediment from the bends generating elevated deposits dissected by small gullies. Matsubara et al. (7) noted high solute concentrations and clear evidence of flocculation of the mud being carried in suspension. They also documented mud aggregates forming ripples and dunes. The mean channel slope is about 1.5 ×$10^{-4}$, bankfull discharge about 11.9 $\;{\text{m}}^{3}\;{\text{s}}^{-1}$ with a width of 20 m and depth of 1.1 m.

## Modeling Lateral Accretion Deposition

We use the Delft3D model (e.g. ref. 27) to investigate the hydro-morphodynamics of the Quinn River. In this model, the transport of sand and mud is often treated separately for the case of mixed bed of sand and mud. Previous research (e.g. refs. 28–32) demonstrate that when the proportion of mud on the bed exceeds about 10%, the mixed sediments exhibit cohesive properties that control the entrainment of sand from the bed. Given the high mud content in the Quinn River samples (7) and that nearly all the sand is less than 0.2 mm, we elected to include sand in the model of cohesive sediment transport. Braat et al. (33) in their model of estuary evolution and mudflat deposition also used Delft3D and treated the fine sand as part of the cohesive sediment transport when the clay content of the bed was significant. They modeled two size classes, sand and mud, and assigned a distinct settling velocity to each. Here, we modeled six size classes: two sizes each of fine sand, silt, and clay size classes, which roughly follow phi scale divisions. We limited the model to six size classes to reduce computational time.

We use the Partheniades–Krone formulations (34, 35) to calculate the erosion and deposition rates of cohesive sediment. Although a simplification of the complex dynamics of deposition and entrainment of muddy sediments, it is widely used in modeling muddy estuarine and tidal systems (e.g. refs. 33, 36, and 37). The entrainment of cohesive sediment from the bed occurs when the local boundary shear stress exceeds the critical threshold. In the case of a mixed grain size sediment bed, the erosion rate of each sediment size class is proportional to its fraction present in bed surface ($p_{i}$). Because we are modeling six grain size classes, we use the subscript (i) on the fraction of the bed, settling velocity, and basal concentration in that size class.

[1] $$\begin{array}{c} E=\left\{\begin{array}{cc} M\left(\frac{\tau_{b}}{\tau_{\mathrm{ce}}}-1\right)p_{i}, & \tau_{b}>\tau_{\mathrm{ce}}\\ 0, & \tau_{b}\leq \tau_{\mathrm{ce}} \end{array}\right., \end{array}$$

Where *E* is the erosion rate for cohesive sediment ($\text{kg}\;\;{\text{m}}^{-2}\;\;{\text{s}}^{-1}$), *M* is a characteristic erosion rate parameter ($\text{kg}\;\;{\text{m}}^{-2}\;\;{\text{s}}^{-1}$), $\tau_{b}$ is the local boundary shear stress (Pa), $\tau_{\mathrm{ce}}$ is the critical erosion shear stress (Pa), $p_{i}$ is the sediment fraction on the surface bed layer for size class i. Here, we do not consider the complex interactions at the bed associated with periodic disruption of the viscous sublayer by turbulent instabilities, changing mobility of sediment postdeposition and burial, effects of temperature, and other factors (e.g. ref. 38).

The deposition rate is given by the product of the settling velocity, the local basal concentration, and the probability of deposition:

[2] $$\begin{array}{c} D=\left\{\begin{array}{cc} w_{\mathrm{si}}c_{\mathrm{bi}}\left(1-\frac{\tau_{b}}{\tau_{\mathrm{cd}}}\right), & \tau_{b}<\tau_{\mathrm{cd}}\\ 0, & \tau_{b}\geq \tau_{\mathrm{cd}} \end{array}\right., \end{array}$$

Where *D* is the deposition rate for cohesive ($\text{kg}\;\;{\text{m}}^{-2}\;\;{\text{s}}^{-1}$), $w_{\mathrm{si}}$ is the sediment settling velocity (m/s), $c_{\mathrm{bi}}$ is the mass concentration (kg/m^3^), $\left(1-\frac{\tau_{b}}{\tau_{\mathrm{cd}}}\right)$ is the probability that a sediment particle will stick to the bed (e.g. ref. 39), $\tau_{b}$ is the local boundary shear stress (Pa) and $\tau_{\mathrm{cd}}$ is the critical deposition shear stress above which no deposition of sediment will occur (Pa). The probability of deposition term is controversial and has been argued to not be correct and to be excluded from the model (e.g. refs. 33, 39, and 40). We follow that practice and thus write, *D* simply as

[3] $$D=w_{\mathrm{si}}c_{\mathrm{bi}}$$

and note that *D*-*E*> 0 is net deposition.

The suspended sediment concentrations and channel bed samples collected from the Quinn River were used to determine the three parameters, *M*, $\tau_{\mathrm{ce}}$, and $w_{\mathrm{si}}$, in the Eqs. **1** and **3** (*Materials and Methods*). Outer bank erosion was not considered in this model.

We have conducted 10 separate modeling simulations (41). Here, we report on the last five Runs (Runs 5 to 9); all were done at bankfull discharge (*SI Appendix*, Table S1). We varied the upstream input of mud, and the settling velocities assigned to the mud fraction in successive Runs. The model was applied to 2,300 m long reach for which we had Light Detection and Ranging (LiDAR) topographic data. Field measurements provided higher topographic resolution at one sharp bend (Howard’s Bend Fig. 1) and local data on the grain sizes of lateral accretion deposits (*Materials and Methods*). We report the results mostly for this bend.

A major decision was how to assign a sediment load by grain size at the upstream boundary condition. For Runs 5, 7, and 8, we calculated the sediment concentration for six size classes that would be in equilibrium with the mean boundary shear stress across the 2,300 m long reach (Fig. 1*D*) for the average bed grain size distribution assigned as the initial condition (and derived from field measurements *Materials and Methods*). Setting Eq. **1** equal to Eq. **3**, we solve for the near-bed concentration

[4] $$c_{\mathrm{bi}}=\frac{M\left(\frac{\tau_{b}}{\tau_{\mathrm{ce}}}-1\right)p_{i}}{w_{\mathrm{si}}}$$

In Runs 5 and 7 we assigned settling velocity values of 0.34 mm/s to the fine silt and clay size fractions to account for flocculation (e.g. ref. 22). The fine silt and clay settling velocities in Run 8 were reduced to match settling velocities measured from our field samples (*Materials and Methods*; *SI Appendix*, Table S2). In Run 9 the settling velocities were set equal to rates of individual particles (no flocculation) but we kept the sediment concentration prescribed for Run 5. A brief rainstorm on the Quinn River during our field work generated local mud-rich sheet runoff from exposed Lake Lahontan sediments. This increased concentrations in the channel and led to mud deposition along the channel banks (*SI Appendix*, Fig. S7). Run 6 explores what the depositional consequence would be from increasing the mud load by a factor of 4. The total load at the upstream section is derived from the computed basal concentrations for the six size classes. For silt and clay, the suspended load concentration is predicted in Delft3D to vary little from $c_{b}$ to the surface, hence $c_{b}$ is representation of the average concentration for these grain sizes. For sand, however, the concentration declines significantly above the bed, hence our total load is based on the vertical integral of the concentration profiles for the two sand size classes. Runs 5, 7, 8, and 9 represent five days of constant sediment supply and discharge; Run 6 represents two days.

## Mud Deposition

**Fig. 2.**
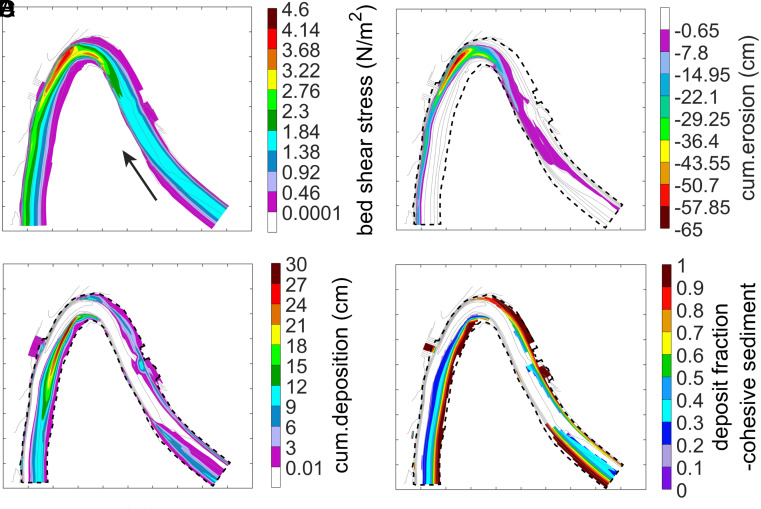
Modeled bankfull boundary shear stress (*A*), erosion (*B*), cumulative deposition (*C*), and fraction of the total cumulative deposit consisting of cohesive size sediment (fine silt and clay) (*D*) for five-day duration of Run 5. Mean boundary shear stress was 1.38 Pa. Sediment input was established to be at equilibrium capacity for six size classes at the mean boundary shear stress. The black dash line represents channel boundary. Grid scale spacing marking the outline of each plot is 20 m. The black arrow indicates the flow direction.

Delft3D predicts that as flow approaches Howard’s bend, due to upstream curvature effects, the high velocity core and boundary shear stress lie near the inner bank (Fig. 2*A*). Through the apex there is rapid curvature change, strong centrifugal accelerations (that leads to steep cross-stream pressure gradients), and shoaling [that leads to convective accelerations (topographic steering)]. Collectively this causes local flow acceleration, increased boundary shear stress and a rapid downstream shift of the high velocity and boundary shear stress locations to the outer bank and the local pool. Strong near-bed inward velocities and suspended sediment flux develop across the apex and just downstream of the apex (*SI Appendix*, Figs. S8 and S9), and then progressively decline in magnitude further downstream in the low curvature lower arm of the bend. A relatively spatially constant boundary shear stress field develops along the downstream inner bank limb of the bend, where lateral accretion deposits extend downstream. This is a characteristic pattern of velocity and boundary shear stress through river meanders that has been documented in the field and laboratory and explained from theory and numerical modeling (e.g. refs. 42–46). All the model Runs reported here show similar velocity and boundary shear stress fields.

In Run 5, net erosion tended to occur where the boundary shear stress was higher than the mean along the outer bank and net deposition approximately tracked the region where boundary shear stress was lower than the mean along the inner bank (Fig. 2*A*–*C* and *SI Appendix*, Fig. S9). Along the inner bank of Howard’s Bend, the fraction of deposited sediment consisting of cohesive sediment (fine silt and clay) progressively increased from less than 10% in deep water to 100% along the shallow water of the bank (Fig. 2*D*). This lateral variation arises from the dynamic adjustments of the cross-stream decline of $\tau_{b}/\tau_{\mathrm{ce}}$, the enrichment of sand from scour in the pool of the original sandy bed, the influence of secondary circulation, and the shoaling of water along the inner bank (*SI Appendix*, Fig. S10). Given our sediment input was designed to be in equilibrium with the mean boundary shear stress (1.38 Pa, i.e. 3.45 $\tau_{b}/\tau_{\mathrm{ce}}$) (Eq. **4**), the expectation was that where shear stress dropped below the mean, deposition would prevail. Scour into the initial bed material, which consisted of 44% sand, and secondary circulation transport toward the center of the channel (*SI Appendix*, Fig. S10), however, elevated the basal concentration of the 0.1 mm sand by nearly two-fold (*SI Appendix*, Fig. S11). Consequently, net deposition of sand was initiated at 1.64 Pa, i.e. $\tau_{b}/\tau_{\mathrm{ce}}$< 4.1, which was farther toward the outer bank where initially there was net erosion of finer particles. This deposition of sand coarsened the bed, increasing its fraction in the bed, $p_{i}$, but thereby reducing $p_{i}$ of the finer sediment. This lower $p_{i}$ reduced the entrainment rate of finer sediment (Eq. **1**) leading to net deposition of mud in areas also where the shear stress was above the mean value. Progressive cross-stream decline in $\tau_{b}/\tau_{\mathrm{ce}}$, and the reduction in sand $c_{\mathrm{bi}}$ due to reduced cross-stream transport led to relative increase in cohesive fraction of the bed deposit. Near the inner bank, the water shoals, $\tau_{b}/\tau_{\mathrm{ce}}$ drops below 1.0 ($\tau_{b}<$ 0.4 Pa) (i.e. no entrainment), cross stream transport ceases, sand drops significantly, and mud dominates the deposit. *SI Appendix*, Fig. S12 shows the fraction of the deposited bed sediment for each of the six size classes.

Commonly it is proposed that a scale for the tendency of suspended sediment to be deposited is the ratio of the settling velocity to shear velocity, typically recorded in the widely cited Rouse number (e.g. refs. 47–52).

[5] $$P=\frac{w_{s}}{\kappa u_{*}}$$

Here, $u_{*}=\sqrt{\frac{\tau_{b}}{\rho }}$, ρ is fluid density, and, κ, the von Karman constant is assumed to be 0.41 (e.g. ref. 53). Where *P* is less than some threshold value, it is proposed that sediment will not deposit into the bed (i.e. washload). Published values vary from 0.8 (48), to 0.25 (47) to as low 0.06 (49). By contrast, in Run 5 significant deposition occurs even when *P* is less than 0.01.

The ratio of deposition to erosion based on Eqs. **3** and **1**, illustrates why *P* is a poor indicator of the tendency for deposition in muddy cohesive controlled sediment transport:

[6a] $$\frac{D}{E}=\frac{w_{\mathrm{si}}c_{\mathrm{bi}}}{M\left(\frac{\tau_{b}}{\tau_{\mathrm{ce}}}-1\right)p_{i}},$$

Where *D* exceeds *E*, net deposition is predicted. Deposition is not represented by the ratio of settling velocity to shear velocity, but rather by the product of setting velocity times sediment concentration near the bed, relative to the magnitude of the boundary shear stress. The value of $p_{i}$ is an evolving composition in a dynamic exchange layer (i.e. *SI Appendix*, Fig. S13).

Note that for the cohesive sediment, the settling velocity in Run 5 was assigned a flocculation-driven settling velocity of 0.34 mm/s. The parameter *M* is fixed for all model Runs at $1.27\times 10^{-4}$$\;\text{kg}\;\;{\text{m}}^{-2}\;\;{\text{s}}^{-1}$ (*Materials and Methods*).

Hence, we can rewrite Eq. **6a** as

[6b] $$\frac{D}{E}=\frac{2.7c_{\mathrm{bi}}}{\left(\frac{\tau_{b}}{\tau_{\mathrm{ce}}}-1\right)p_{i}}$$

This shows that for a given boundary shear stress relative to the critical erosion threshold, and size fraction in the surface bed ($p_{i}$), there is a concentration that will lead to deposition, even for very small settling velocities. While flocculation will favor deposition, the basal concentration can vary by orders of magnitude as well and drive deposition. Furthermore, the smaller the $p_{i}$ for a given grain size, the more likely *D*>*E*.

Run 8 was executed to explore this simple interpretation. In this case, we reduced the settling velocity of the fine silt and clay based on analyses we did of suspended sediment collected in the field (*Materials and Methods*). Eq. **4** was then used to calculate the sediment input for each of the six size classes. The reduced settling velocities of the fine silt and clay leads to higher calculated equilibrium input basal concentrations (*SI Appendix*, Table S2). For exactly the same discharge, initial bed grain size and topography, and model duration, Run 8 predicts the nearly identical boundary shear stress field, patterns of erosion and deposition, and, importantly, the same mud-rich lateral accretion deposits (*SI Appendix*, Fig. S14). In this case, the Rouse number *P* values for the mean boundary shear stress for fine silt, coarse, and fine clay were 0.015, 0.001, and $1.1\times 10^{-5}$, respectively.

In Run 5, the total input load was equal to 409 mg/l, a relatively low concentration for a muddy river, which commonly occurs in muddy rivers [e.g. the Red River (54), the Little Wabash, and Little Sugar Rivers (55)]. In Run 8 the increased sediment load was equivalent to 12,948 mg/l. Such rivers as the Yellow River (e.g. ref. 56) and the Rio Puerco (57) carry this load (and more), though additional effects of density stratification become important (e.g. ref. 58).

This shows that Eq. **4** can be used to evaluate what the sediment load, per grain size, that would be needed for a given settling velocity to cause net sediment deposition of mud in areas of boundary shear stress below the average. If this sediment load is less than equilibrium, deposition is less likely. The extreme case (Run 9) was to assign settling velocities equal to single particles settling in still water (*SI Appendix*, Table S2). If we had used equilibrium values calculated from Eq. **4**, the total load would have been 74,462 mg/l, which does occur in some rivers (e.g. refs. 56, 57, and 59). In Run 9, however, while we reduced the settling velocity to single particles for the mud, we kept the input equivalent to the load for Run 5, where the setting velocities were greatly elevated due to flocculation. The results (*SI Appendix*, Fig. S15) showed the bed fraction of sand was greatly elevated, fine silt greatly reduced, and clay essentially absent. This confirms the role of both settling velocity and sediment concentration have in contributing to deposition rates and that flocculation as often argued contributes to mud deposition.

## Inclined Heterolithic Stratigraphy Due to an Elevated Mud Load Event

In Run 6, the only change relative to five-day Run 5 was the cohesive sediment load (fine silt and clay) was increased by a factor of 4. The bankfull discharge remained constant. This was meant to simulate the consequence of a rainstorm driving mud-charged runoff into the Quinn River from adjacent muddy plains, without a significant change in runoff to this channel draining a large area (as we observed during field work).

**Fig. 3.**
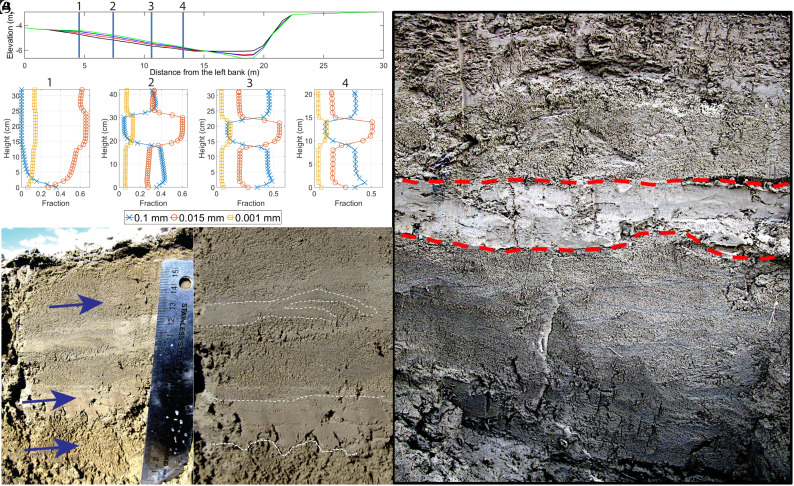
Generation of inclined heterolithic stratigraphy. (*A*) Modeled sand, silt, and clay deposition at a cross-section in Howard’s Bend due to the successive Runs 5, 6, and 7 (The surface topography at the end of runs are represented by red, blue, and green lines). In Run 6, the fine silt and clay load was increased by a factor of 4 relative to Runs 5 and 7. The duration of Runs 5 and 7 was five days each, while Run 6 lasted two days (location of modeled cross-section is shown in Fig. 1*A* and *SI Appendix*, Fig. S9). (*B*) sandy mud interbeds in muddy sand deposits, arrows point to location of samples collected. The *Right* image is a close-up of the *Left* with dashed lines added to denote the *Lower* and *Upper* bounds to the two sandy mud interbeds. (*C*) 2 to 4 cm thick light toned mud-rich interbed in muddy sand deposit.

Initial conditions for Run 6 were the topography and bed material generated Run 5. We modeled Run 6 for 2 d, then followed that with a five-day Run 7, under the same conditions as Run 5, but built on the results of Run 6. This sequence of three Runs generated a distinct, sandy mud layer, similar in size distribution and thickness to that observed in field trenches (Fig. 3*A* and *SI Appendix*, Fig. S16). After a small amount of deposition at the start of each Run (other than in profile 1), the grain size distribution of the deposit remained constant as deposition continued (Fig. 3). This constant size distribution during aggradation reflects the relative constancy of arriving sediment concentrations and local boundary shear stress. Other than the small adjustment in the bed size fraction ($p_{i}$), all the terms in Eqs. **1** and **2** were invariant. Similarly, with the introduction of a large mud supply, bed fining developed until a new constant deposition rate developed, creating a near constant grain size deposit recording the mud pulse. In Run 7, upon returning to the sediment load of Run 5, the deposited sediment changed back to the same relative sediment concentrations and surface grain sizes, resulting in a deposit of nearly identical composition of Run 5. The sharp bottom and top boundaries model results also closely resembled sandy mud interbeds observed in various lateral accretion trenches on the Quinn River (Fig. 3). In the shallow water along the inner bank, little sand arrives, consequently, the mud event is expressed as small increases in silt and clay. The distinctive heterolithic sequence, then, did not extend into the shallow water area.

## Predicted Grain Size Distributions Compared to Field Data

Matsubara et al. (7) reported 132 grain size analyses ranging from the Lake Lahontan deposits, bank exposures, trenches, cutoff deposits, and the floodplain, as well as many grab samples from unspecified locations. We replotted their graph, eliminating the 71 samples from unspecified locations and added five channel bed material samples (that formed the basis of the initial bed grain sizes in the model), 8 samples from Howard’s bend lateral accretion to scroll bar deposits, and 1 sample from the deposit created by a storm even during our field work (*SI Appendix*, Fig. S17).

**Fig. 4.**
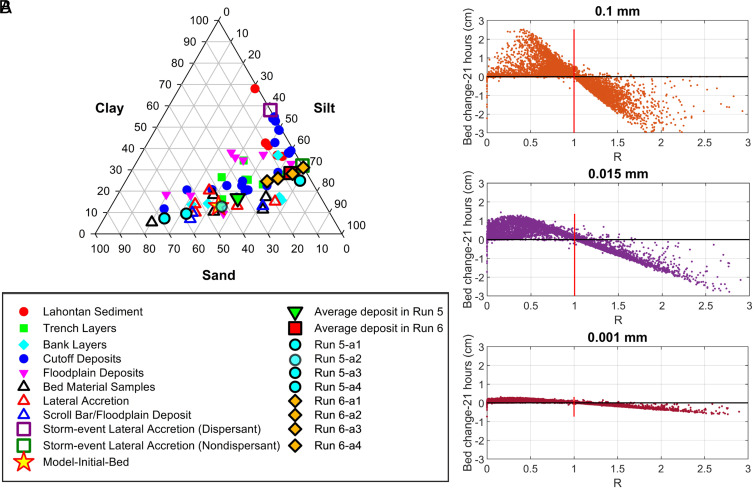
(*A*) Comparison of predicted grain size distribution across the Howard’s Bend lateral accretion deposit with field samples reported by Matsubara et al. (7) and collected in our field study. Runs 5 and 6 (a1 to a4) results shown in Fig. 3*A*. (*B*) deposition (>0) and erosion (<0) over a 21-h period compared to *R*, a dimensionless ratio of local boundary shear stress to the equilibrium value (no erosion or deposition). *R* for a specific grain size depends on the settling velocity, basal concentration, and local size fraction in the surface bed material.

Fig. 4*A* compares model predictions of the grain size distribution of deposits with field observations. The predicted vertical grain size variation in two cross-sections across the lateral accretion deposits generated by Runs 5 and 6 are similar and for each Run are nearly constant throughout the deposition period (Fig. 3 and *SI Appendix*, Fig. 16). Consequently, average deposits of Run 5 and Run 6 are assessed for each of the four profiles across a representative cross-section. In addition, the average grain size properties of the entire 2,300 m lateral accretion deposits are plotted for each of these two Runs. All the models of grain sizes match field data. It is worth noting that the plots were constructed after completion of all the Runs. No attempt to adjust or calibrate parameters to meet the data was attempted.

## Discussion

By employing Eq. **4** to establish in upstream sediment supply we created a condition in which for a spatially constant mean boundary shear stress there would be no net deposition or erosion: The flow would carry the load at capacity. Einstein et al. (60) introduced the term “wash-load” to describe sediment that bears no relationship to discharge and is washed through the system. Einstein and Chien (20) then stated, “wash load” occurs when supply of these particles is “permanently below the capacity of the stream to move them.” They did suggest that transient surficial deposition of wash load on the bed can occur, but that it is unpredictable, and in general wash load does not occur in significant quantity in the bed. Wash load is controlled by supply to the river, and cannot be calculated from bed material composition. The importance of load relative to capacity has been emphasized by others (e.g. refs. 21, 51, and 61). By introducing sediment supply at capacity for the mean boundary shear stress in our models, the topographically driven shifting high and low boundary shear stress fields, led to patterns of erosion and deposition corresponding to sediment loads below and above capacity.

The nondimensional ratio of local boundary shear stress to the equilibrium value for the local settling velocity, basal concentration, and surface bed fraction can be written as

[7a] $$R=\frac{\tau_{b}}{\tau_{\mathrm{ce}}}\left(\frac{1}{\frac{w_{\mathrm{si}}c_{\mathrm{bi}}}{Mp_{i}}+1}\right),$$

Where *R* > 1 predicts erosion and < 1 deposition. In Fig. 4*B*, the *R* value for all 14,972 cells representing the bed of the 2,300 m long reach is plotted versus the local erosion or deposition over a 21-h period of Run 5. Local values of $c_{\mathrm{bi}}$ and $p_{i}$ were used. The pattern of *R* predicts in nearly all cases the observed tendency for erosion and deposition, and, importantly, only a small number of cells occur exactly at equilibrium (no erosion or deposition).

As was done for Eq. **4b**, using the same fixed values for $w_{\mathrm{si}}$ and *M*, Eq. **7a** can be written as

[7b] $$R=\frac{\tau_{b}}{\tau_{\mathrm{ce}}}\left(\frac{1}{\frac{2.7c_{\mathrm{bi}}}{p_{i}}+1}\right)$$

This highlights that the spatially varying concentration, $c_{\mathrm{bi}}$ and bed surface grain size fraction, $p_{i}$, play a significant role in whether deposition or erosion occurs. Settling velocity does matter: With increasing settling velocity, *R* would decline and deposition would be more likely.

This analysis also then further argues against the utility of relying on the assumption that $w_{s}/u_{*}$, as expressed in the Rouse number (Eq. **5**), indicates a tendency for mud deposition. The Rouse number, *P*, results from the solution for the prediction of an equilibrium profile in which downward advection of sediment, scaled by the product of settling velocity and basal concentration is balanced by upward diffusion of sediment due to the local turbulence (scaled by the shear velocity) from a defined near bed concentration (62). *P* is the exponent on the term for height above the bed that predicts the corresponding suspended sediment concentration. In essence, *P* predicts the concentration profile, not the deposition rate of sediment on the bed. The assumption has been that where the concentration profile is vertically constant, there is no net flux to the bed. In the case of deposition in muddy rivers, in which supply is controlled by upstream fluxes, the Partheniades–Krone criteria, expressed in Eqs. **3**, **4**, **6**, and **7**, shows that it is the spatially varying $w_{s}c_{b}$ relative to the $\tau_{b}/\tau_{\mathrm{ce}}$ and $p_{i}$ that determines deposition. While flocculation clearly increases deposition rates, significant mud deposition can occur even when settling velocity is orders of magnitude below shear velocity.

Comparing the grain size distribution of the input, the net deposition, net erosion, and output for the 2,300 m long reach shows that due to erosion of the sandy original bed material, 6 times more 0.1 mm sand (and nearly 40 × more 0.22 mm) was deposited in the lateral accretion deposits than was supplied at the upstream boundary condition (*SI Appendix*, Table S3). Hence, the tendency for sand to deposit relative to mud, will lead to a sustained sandy deposits as rivers shift across their floodplains, even when the upstream supply is relatively slow.

While we propose that the model results presented here explain sediment transport mechanics driving lateral accretion in muddy meanders, much more can be explored within this model framework. In our model, the critical shear stress is a spatial and temporal constant, which is unlikely in natural channels. Deposited mud experiences consolidation, increasing the critical shear stress for entrainment (e.g. refs. 36 and 63), and seasonally exposed dry banks and beds may further strengthen the mud. It may be, for example, that scour depth in pools may be limited by elevated $\tau_{\mathrm{ce}}$ values as more deeply deposited sediments are exposed. We used a constant discharge and sediment supply over a five-day period. While on very large rivers long periods of near constant discharge and sediment load develop, most rivers are driven by storm events and fine sediment load may covary, and, commonly, are hysteretic (64). Given the evidence that just an increase in mud concentration can lead to a characteristic mud interbed member of the inclined heterolithic stratigraphy of meandering rivers, it will be instructive to explore the stratigraphic record that would be produced by discharge dynamics and duration and magnitude of change in mud events. Is there a characteristic stratigraphic signal due just to a mud concentration change? Floodplain deposition is significant on the Quinn River, and its role in mud accretion and stratigraphic evolution of the deposits should be explored, including levee formation. Ultimately, linking the model to a bank erosion model would then allow exploration of the long-term morphodynamics and stratigraphic record of muddy meandering rivers. Braat et al. (65) explores the effects of gravity differences between Earth and Mars, pointing out that settling velocities will be less, and for the same stream flow depth and slope, the boundary shear stress will be less. They did not consider muddy cohesive sediment transport. This can now be explored with this model. Our investigation opens the doors to many questions about muddy meander morphodynamics, stratigraphic signatures, and behaviors on other planets.

## Materials and Methods

The three model parameters, $p_{i}$, $\tau_{\mathrm{ce}}$, and *M*, were determined from field measurements and literature review. The fraction of the bed surface in a particle size class, $p_{i}$, is only a parameter for the initial bed of the channel. Once erosion and deposition occur, $p_{i}$, becomes a response variable. At an upstream relatively straight reach, at five locations across an 11 m wide bed, we collected 1 cm thick samples of bed material using an Eckman sampler. Walking on the soft bed and insertion of a rod found the bed to be of modest density to a depth of typically 10 cm. The collected samples were treated with dispersant and analyzed using a Malvern^®^ Mastersizer^®^ 3000. A single average grain-size distribution, divided into six size classes, was used to define the $p_{i}$ values for the initial bed (*SI Appendix*, Table S2). The critical boundary shear stress for erosion, based on the relatively high proportion of fine silt (27.5%) and clay (13%) [and total of 57% less than sand (63 μm)] in a modest density deposit, was assigned a value of 0.4 Pa (32). This value falls within the range of values estimated in 12 estuarine studies summarized by Tao et al. (37) and is acknowledged to be difficult to determine from physical properties of the sediments and deposits (e.g. refs. 36 and 66). To determine *M*, we collected suspended sediment samples at the same cross-section of the bed material sampling (hence $p_{i}$ is known), and ran Delft3D and the Partheniades–Krone model to steady state for a reach across the section, systematically varying *M* to obtain a best fit to the measured suspended sediment concentrations through the profiles. Our sampling was at low flow and relied on three suspended sediment profiles providing us with nine concentrations for each of the size classes (*SI Appendix*, Fig. S18). We size analyzed samples both with and without dispersant for suspended sediment analysis but relied here on the dispersed size distribution to define concentrations per size class and computed sediment settling velocities from ref. 67. Given the strong upstream control on the clay size fraction, we only used the sand and silt size classes. The best fit *M* value was $1.27\times 10^{-4}$$\;\text{kg}\;\;{\text{m}}^{-2}\;\;{\text{s}}^{-1}$, a value within commonly selected ranges for this parameter (e.g. refs. 33 and 37).

The size analysis of untreated and dispersed suspended sediment samples differed significantly for the fine silt and clay sizes, indicating a significant role of flocculation (as noted by Matsubara et al. (7), and likely driven by elevated salt in the water). But our samples were disturbed in transit and may underestimate the strength of the flocculation. Consequently, in conducting our model Runs, we assigned single grain size settling velocities to the sand and coarse silt, and followed the recommendation of ref. 22 by assigning a value of 0.34 mm/s to the fine silt and clay (ranging from 2.7 to 1,236 times faster than single grain size values). In Runs 5 to 7 we use these values but in Run 8 we use an estimate of the settling velocities of these fine sizes based on differences between untreated and dispersed samples, resulting in lower velocities for these sizes, but for clay still well above single grain size velocity values.

Delft3D was run with 10 vertical layers with a logarithmic spacing in the water column for all Runs (going from bottom to surface at 2%, 3%, 4%, 6%, 8%, 10%, 12%, 15%, 20%, 20% depths). The bed layers in the simulation consisted of one “dynamic exchange layer,” referred to as the “transport layer” in the Delft3D model, 400 sublayers, and one base layer (represents the nonerodible base or the depth limit below which erosion cannot occur). Each layer had a uniform thickness of 1 cm. The dynamic exchange layer function is described in the Supplemental Information (*SI Appendix*, Fig. S13). Once flow achieved steady state (after 6 h), erosion and deposition were initiated with the Partheniades–Krone model. The morphological scale factor is set as 1 for all Runs. Time step was 0.01 min per iteration for each model Run (*SI Appendix*, Table S4).

For all Runs, the bed roughness was set equal to the Manning’s n value of 0.027, estimated under 3/4 bankfull discharge in ref. 25. In the simulations, both background horizontal eddy viscosity and horizontal eddy diffusivity were set to $1.0\times 10^{-6}$$\;{\text{m}}^{2}\;{\text{s}}^{-1}$. The κ-ε turbulence closure model was used. In the simulation reach, the upstream boundary was maintained at a constant total discharge throughout the Runs. The downstream boundary was set as a fixed water level.

In *SI Appendix*, Table S3, the total transported mass for input/output over five days was calculated by multiplying the concentration, flux, and the total duration in seconds. The total accumulated change of erosion or deposition was determined by summing the sediment amount change for each pixel over the five-day period. In both calculations, we assumed the same dry bed density (500 $\;\text{kg}\;\;{\text{m}}^{-3}$) to represent the surface layer conditions. Subsequent consolidation and bulk density increase, which follow during burial, was not included in the model.

## Supplementary Material

Appendix 01 (PDF)

## Data Availability

All study data are included in the article and/or *SI Appendix*.
