# Supplementary material for: The sediment transport mechanics driving lateral accretion in muddy meanders
Source: Proc Natl Acad Sci U S A. 2025 Jul 1;122(27):e2506462122. doi: 10.1073/pnas.2506462122 (PMC12268263; doi:10.1073/pnas.2506462122)
Supplement: Supplementary file 1 — Appendix 01 (PDF) [file pnas.2506462122.sapp.pdf]

Supporting Information for

The sediment transport mechanics driving lateral accretion in muddy meanders

Runze Miao, Alan D. Howard, and William E. Dietrich
Runze Miao, William E. Dietrich
Email: miaorunze2011@berkeley.edu, dietrich@berkeley.edu

This PDF file includes:

Tables S1 to S4
Figures S1 to S18
SI References

Table S1. The model settings for simulated cases using Delft3D

#	Discharge (m^3/s)	Day	Sediment supply from upstream (kg/m^3)						Mean boundary shear stress (Pa)	Settling velocity	Note
			0.22 mm	0.1 mm	0.046 mm	0.015 mm	0.003 mm	0.001 mm			
**Bankfull discharge 11.9 m^3/s- Howard Bend simulations** (reach: 2,300 m with river surveyor topo; 4 m bed depth with 1 cm multiple layers)											
**5**	11.9	5	0.00132	0.0208	0.0388	0.249	0.0634	0.0544	1.38	#3	Sediment supply under equilibrium condition
**6**	11.9	2	0.00132	0.0208	0.0388	0.996	0.254	0.217	1.32	#3	4× mud supply after Run 5
**7**	11.9	5	0.00132	0.0208	0.0388	0.249	0.063	0.0544	1.38	#3	Sediment supply under equilibrium condition after Run 6
**8**	11.9	5	0.00132	0.0208	0.0388	0.364	1.477	11.065	1.38	#2	Sediment supply under equilibrium condition
**9**	11.9	5	0.00132	0.0208	0.0388	0.249	0.063	0.0544	1.38	#1	Settling velocity #1 with sediment supply in Run 5

Table S2. The sediments size classes used in Delft3D simulations and the corresponding bed fractions and settling velocities for three different scenarios

	Grain size range(mm)	Representative grain size (mm)	Initial bed fraction	Settling velocity #1 (mm/s)	Settling velocity #2 (mm/s)	Settling velocity #3 (mm/s)
**Sand**	0.3 -0.14	0.22	0.08	18.64	18.64	18.64
**Sand**	0.14-0.063	0.1	0.355	5.26	5.26	5.26
**Silt**	0.063 -0.03	0.046	0.16	1.27	1.27	1.27
**Silt**	0.03 -0.004	0.015	0.275	0.126	0.233	0.34
**Clay**	0.004 -0.002	0.003	0.07	0.00329	0.0146	0.34
**Clay**	0.002 -0.0005	0.001	0.06	0.000275	0.00167	0.34

Table S3. Sediment budget (Input, deposition, erosion, and output amounts) of six sediments in Run 5 (bankfull discharge with equilibrium sediment input and settling velocity #3)

	Input amount (kg)	Deposition amount (kg)	Erosion amount (kg)	Output amount (kg)
**Sand _0.22 mm**	1,696	63,907	63,583	3,107
**Sand _0.1 mm**	48,838	300,041	320,038	80,443
**Silt _0.046 mm**	167,590	153,559	147,033	136,385
**Silt _0.015 mm**	1,280,059	383,182	252,258	1,072,382
**Clay _0.003 mm**	325,927	97,561	64,207	268,465
**Clay _0.001 mm**	279,660	83,705	55,031	230,392
**Total**	2,103,770	1,081,955	902,150	1,791,174

Table S4a. Sediment characteristics in simulations

Sediment property		Unit	Value
**Critical boundary shear stress for erosion**	τ_{ce}	Pa	0.4
**Critical boundary shear stress for deposition**	τ_{cd}	Pa	1,000
**Erosion parameter**	M	kg/m^2/s	1.27×10^{-4}
**Specific density**	ρ_s	kg/m^3	2,650
**Dry bed density**	ρ	kg/m^3	500
**Reference density for hindered settling**	C_{soil}	kg/m^3	1,800

Table S4b. Model parameters for simulations

Parameter	Unit	Value
**Time step**	min	0.01
**Temperature**	ºC	9.2
**Gravity**	m/s^2	9.81
**Water density**	kg/m^3	1,000
**Salinity**	ppt	0
**Initial conditions-concentration**	kg/m^3	0
**Bottom roughness-Manning**	---	0.027
**Background horizontal viscosity**	m^2/s	10^{-6}
**Background horizontal diffusivity**	m^2/s	10^{-6}
**Background vertical viscosity**	m^2/s	10^{-6}
**Background vertical diffusivity**	m^2/s	10^{-6}
**Morphological scale factor**	---	1
**Minimum depth for sediment calculation**	m	0.02
**Transport layer**	m	0.01
**Sublayer**	m	0.01
**Number of sublayers**	---	400
**Spin-up time before morphological changes (for Run 5, 8, 9)**	min	360
**Spin-up time before morphological changes (for Run 6, 7)**	min	0

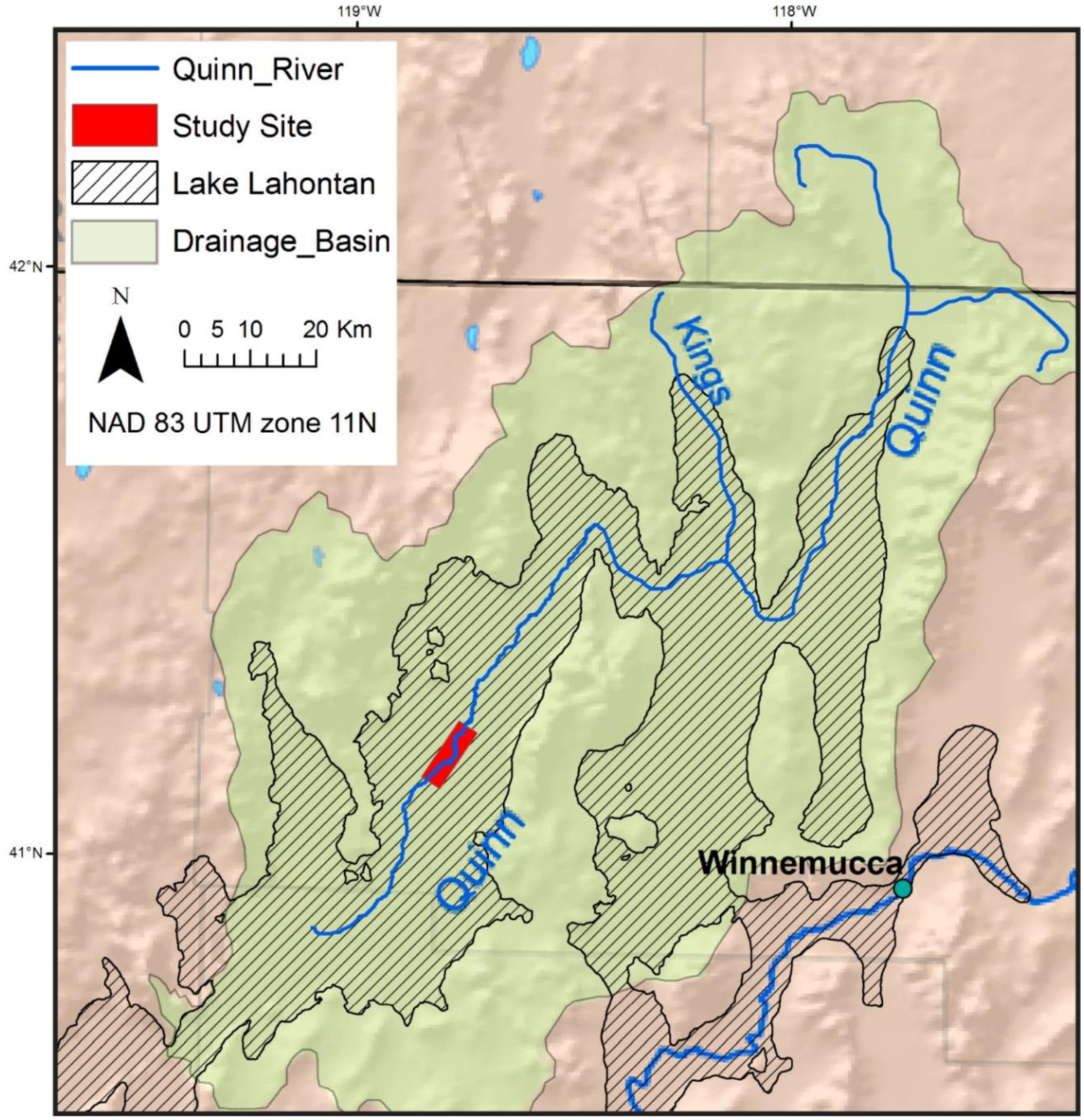

Fig. S1. Red box delineates LiDAR coverage on the Quinn River study area. Green defines the drainage area and the diagonal shading mapped extent of Lake Lahontan (modified from Matsubara et al. (1)). Flow is from the upper right to the lower left.

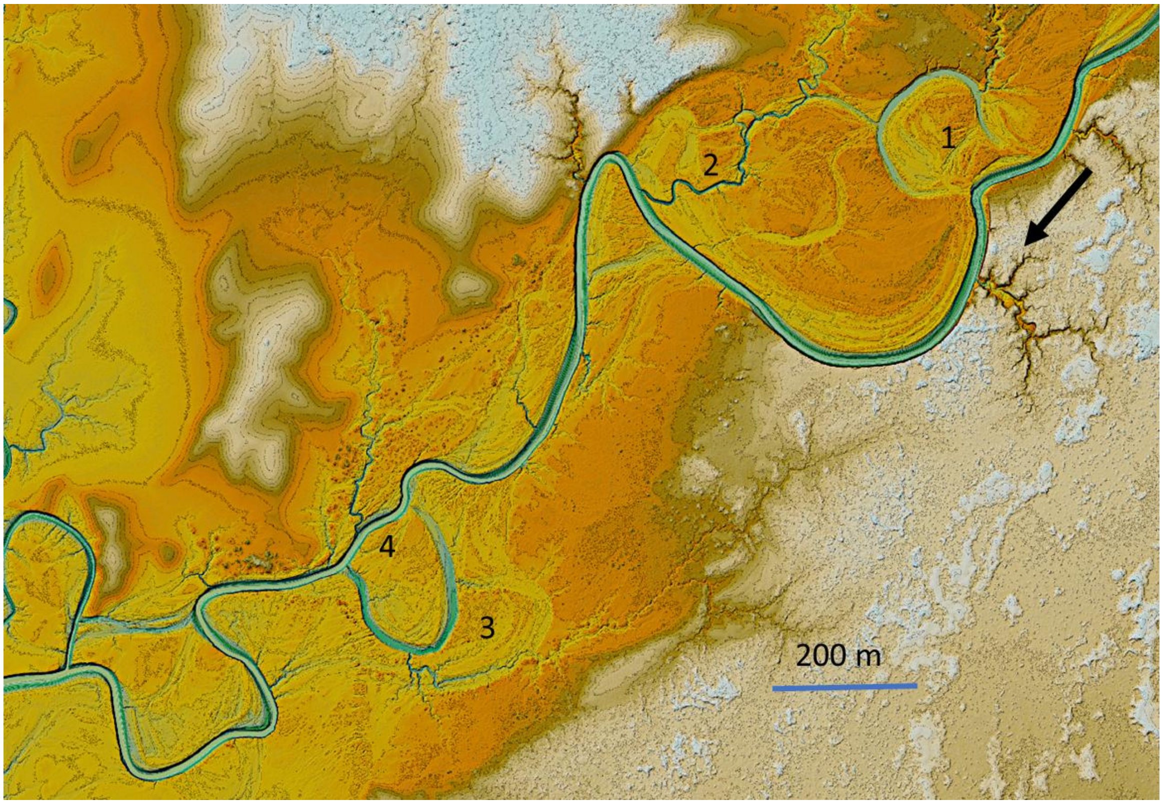

Fig. S2. Colorized elevation of 20 cm contour map of the 2,300 m long reach of the Quinn River modeled with Delft3D. Four bend cutoffs show meander dynamics within floodplain deposits. Convex contours on inner banks of bends delineate lateral accretion deposits. Lake Lahontan sediments occur in the brown to light blue colored topography. The channel bed is colored green. The black arrow indicates the flow direction.

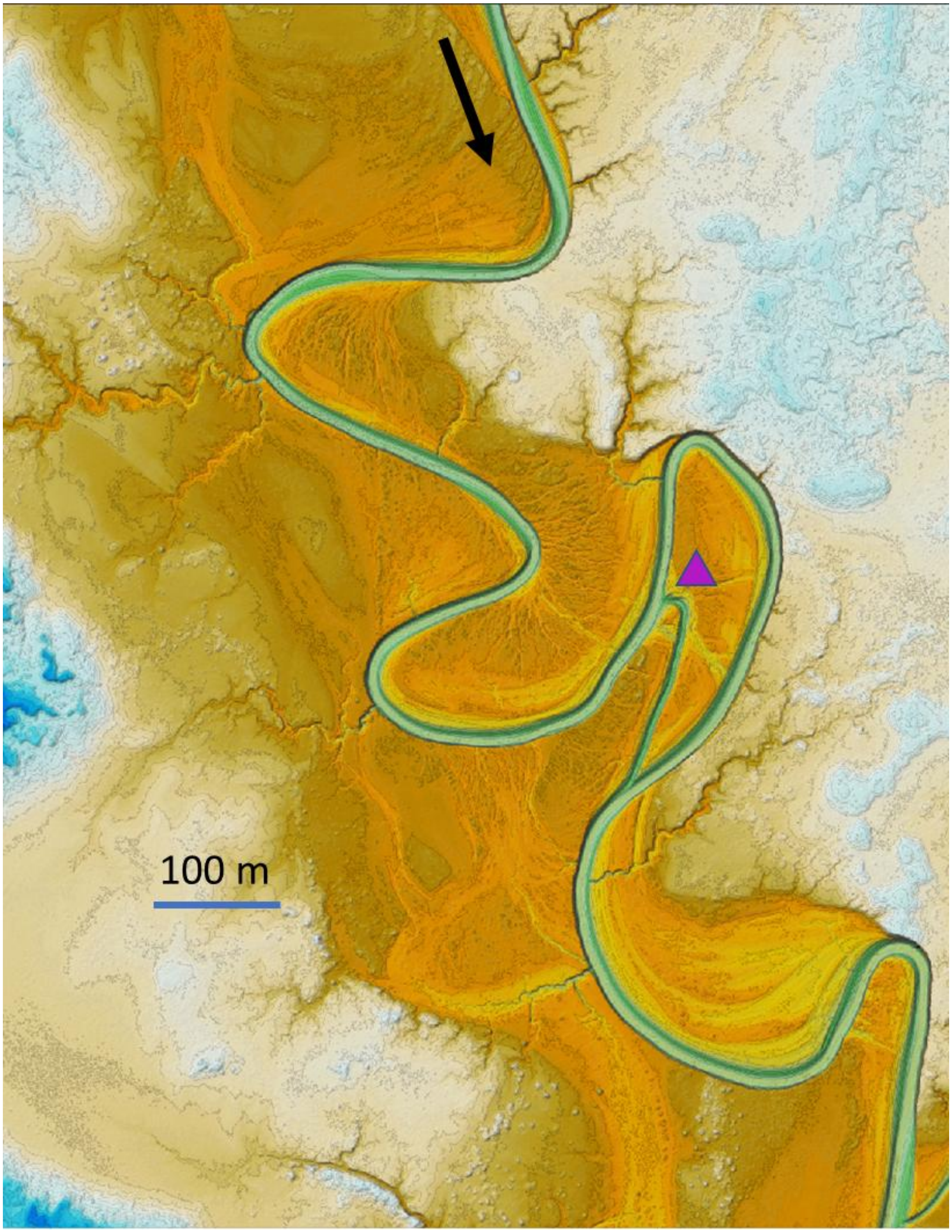

Fig. S3. Colorized 20 cm contour map of a reach downstream of modeled section, showing large amplitude bends, arcuate lateral accretion topography, and radial small channels spreading downslope across the accretionary floodplain. The LiDAR survey was completed in 2010 and by 2020 the chute channel at the triangle cut off the bend. The black arrow indicates the flow direction.

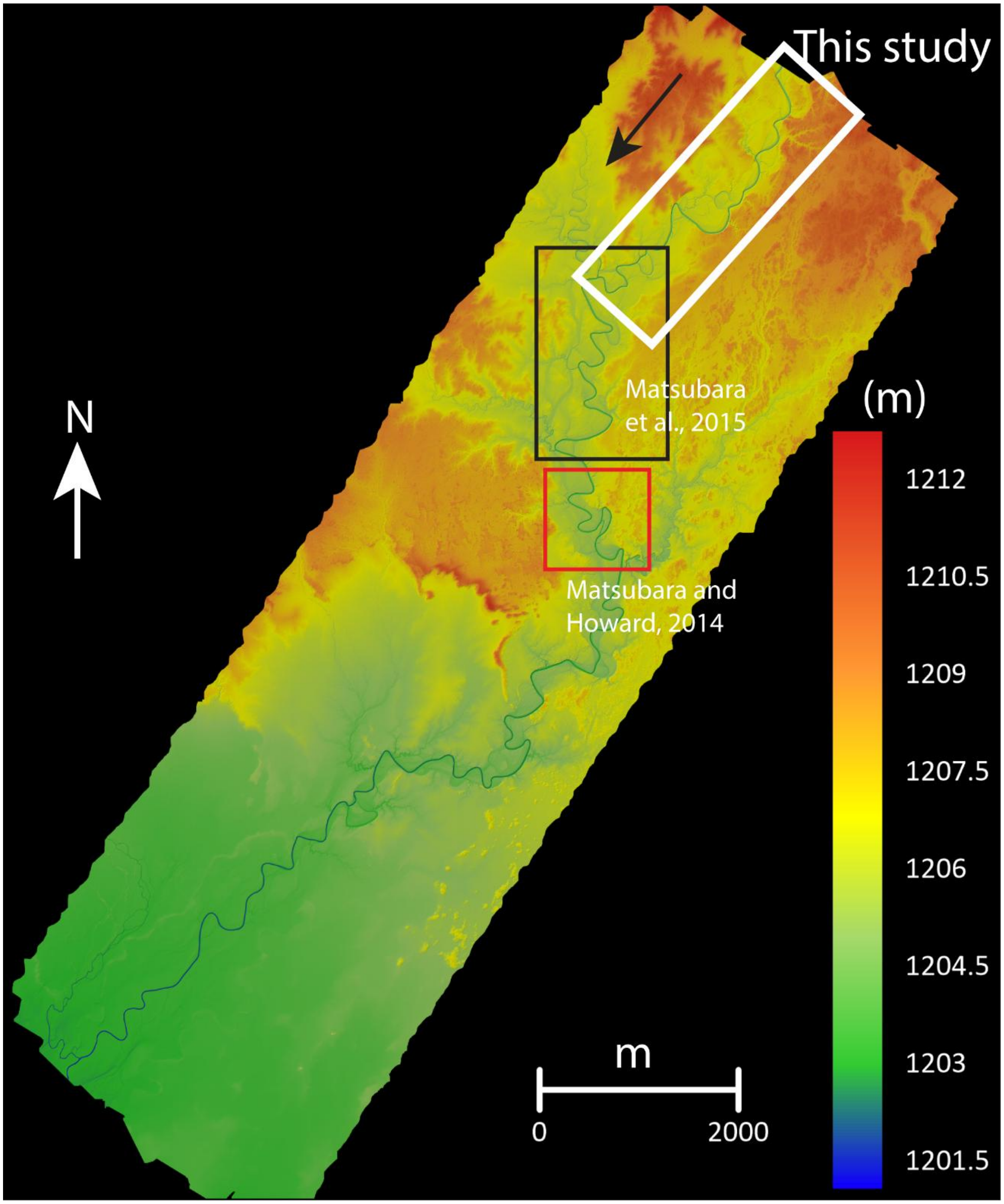

Fig. S4. Location of field and modeling study sites on the Quinn River. The black arrow indicates the flow direction.

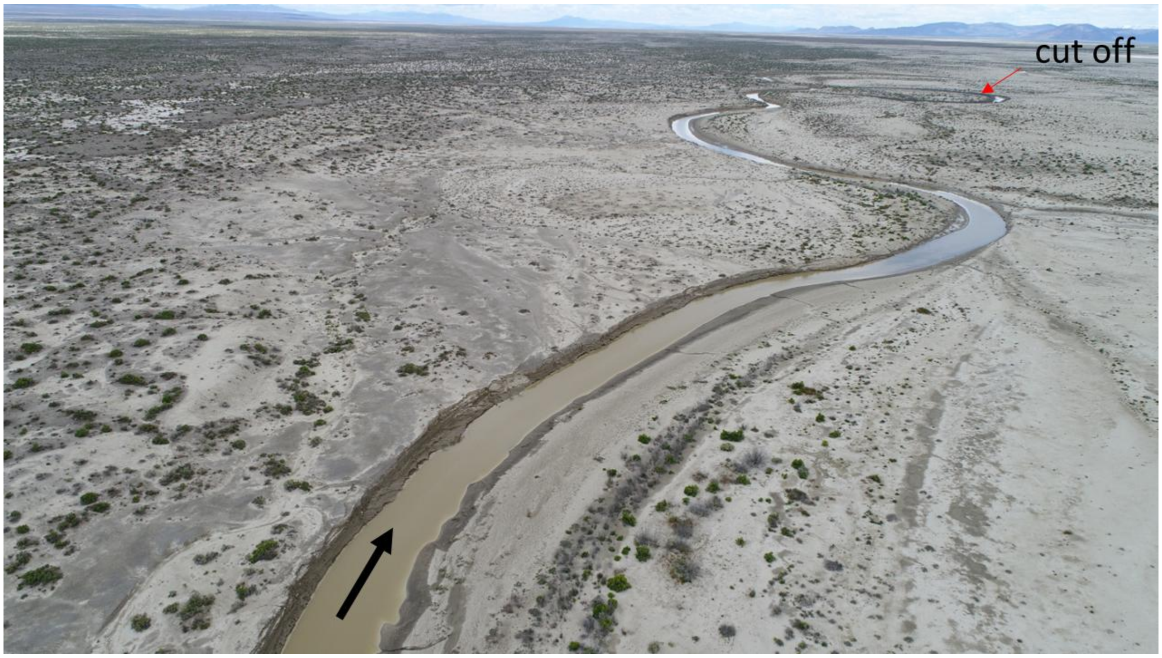

Fig. S5. Drone view looking downstream from close to where bed material and suspended sediment was collected (Luca section) towards cut off number 1 in *SI Appendix,* Fig. S2. Note the low density of vegetation and lateral accretion deposits in the foreground. The black arrow indicates the flow direction.

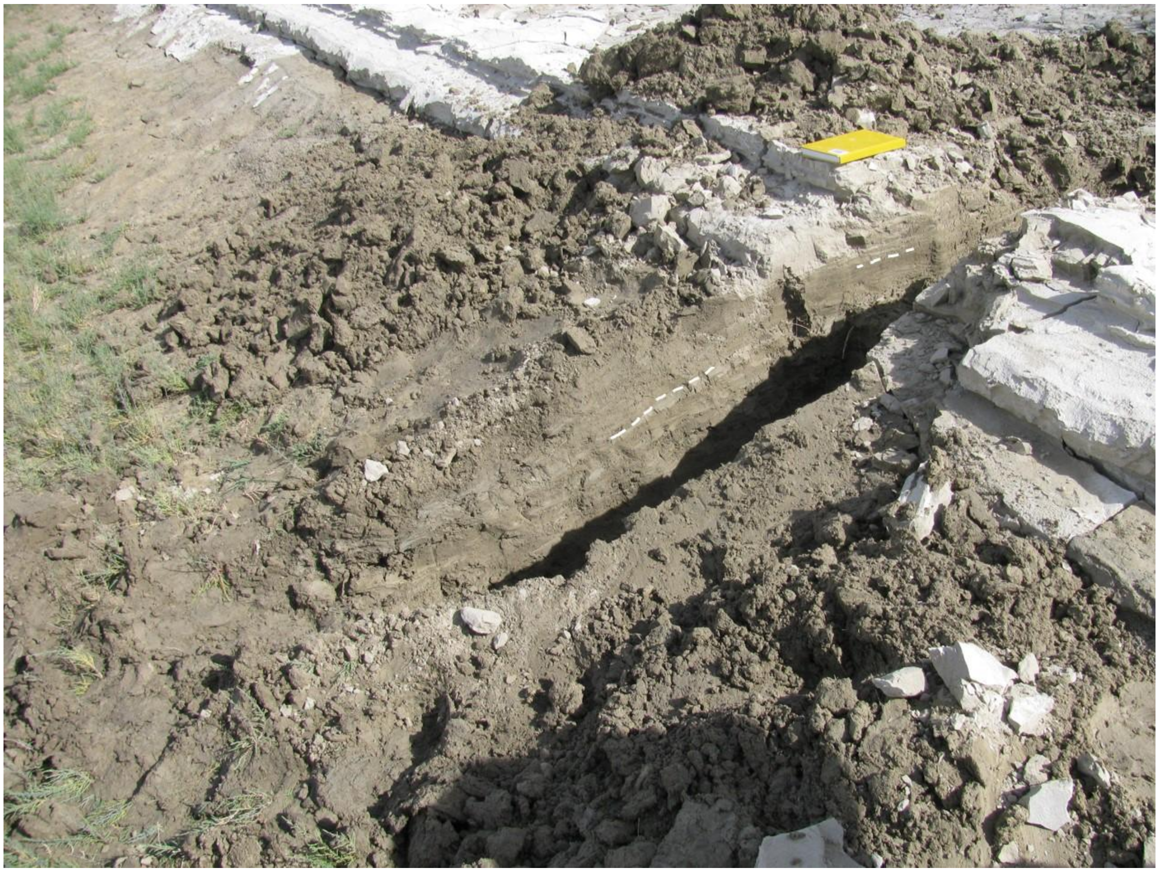

Fig. S6. Trench wall exposure across lateral accretion deposits. Dashed lines track thin, muddier interbeds. Color change reflects surface drying of sediment.

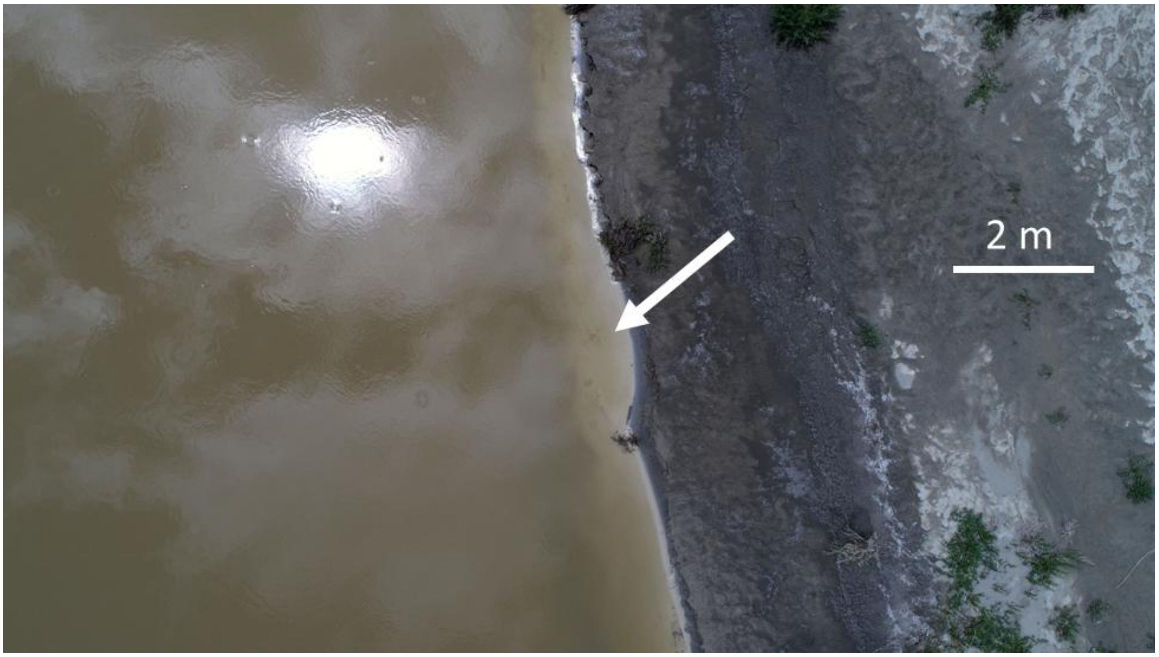

Fig. S7. Drone image of channel bank revealing light toned mud deposit (white arrow) formed in response to a brief storm that elevated suspended concentration by at least 1.6 times. Flow from top to bottom.

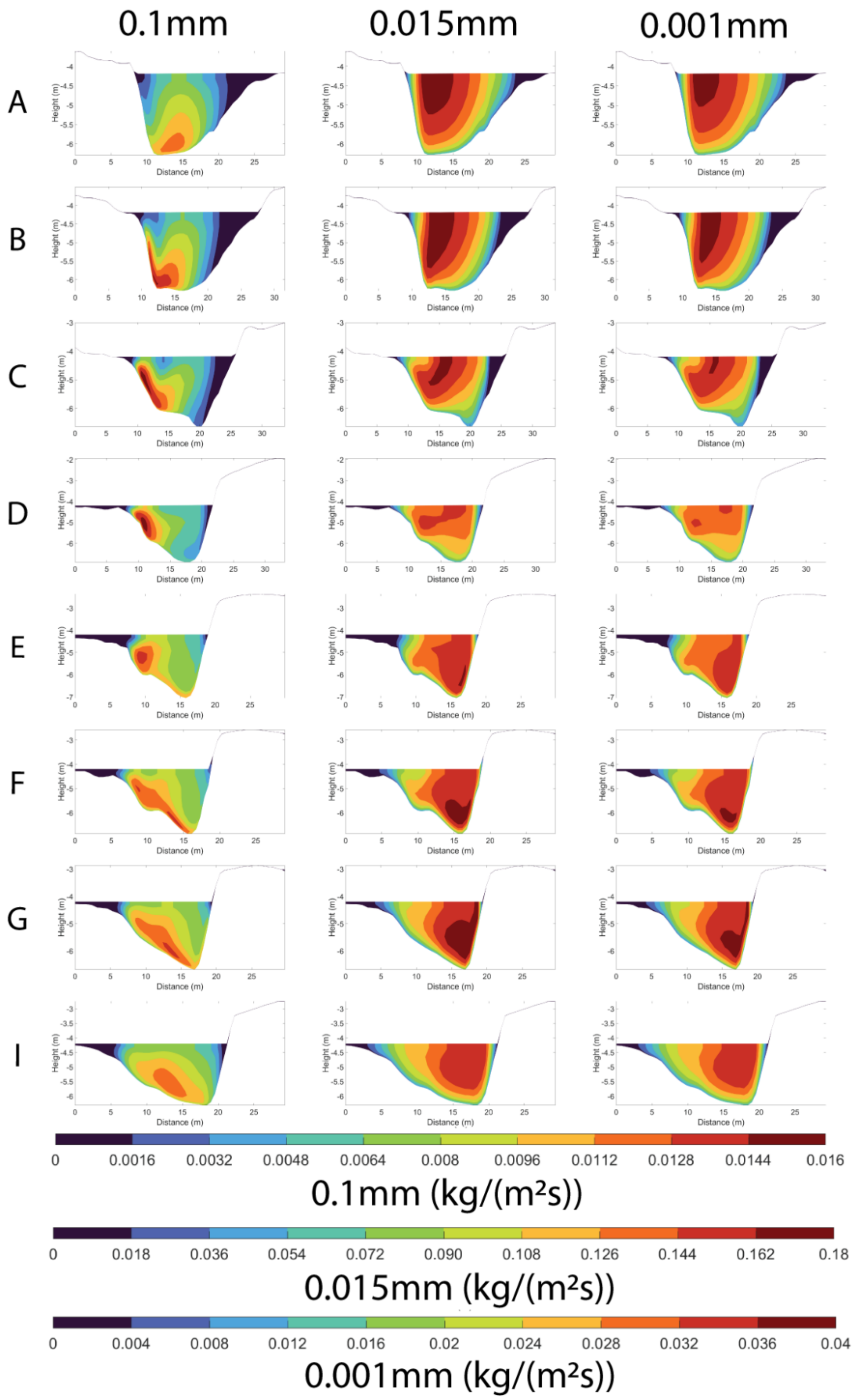

Fig. S8a. Downstream view of modeled downstream sediment transport for three size classes through Howard's Bend in Run 5 (bankfull discharge with equilibrium sediment input and settling velocity #3). Note strong vertical exaggeration and large differences in flux scales for the three size classes.

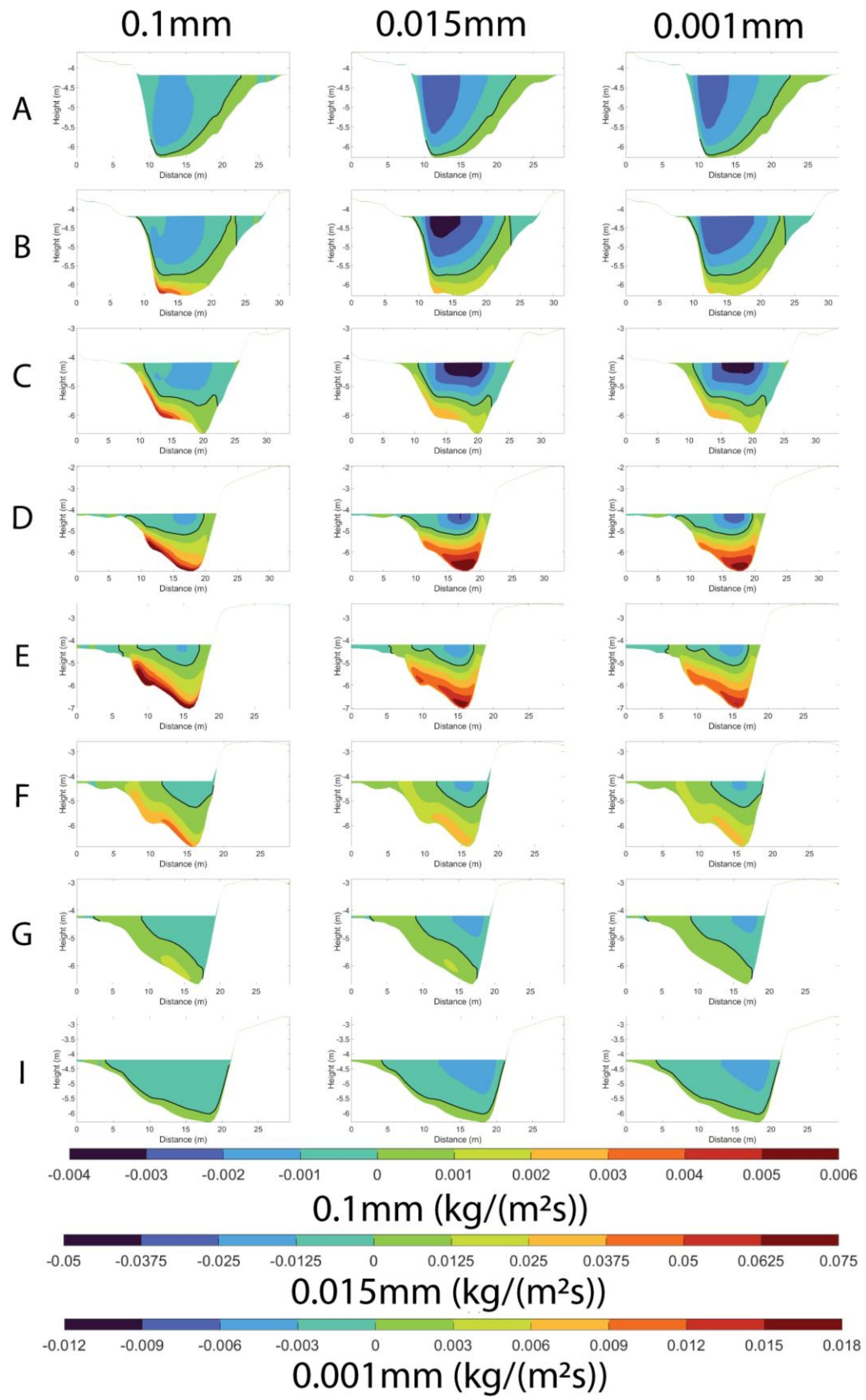

Fig. S8b. Downstream view of modeled cross-stream sediment transport for three size classes through Howard's Bend in Run 5 (bankfull discharge with equilibrium sediment input and settling velocity #3). The positive sign demonstrates flow towards the left bank. The black line divides the fluxes flowing in opposite directions. Note strong vertical exaggeration and large differences in flux scales for the three size classes.

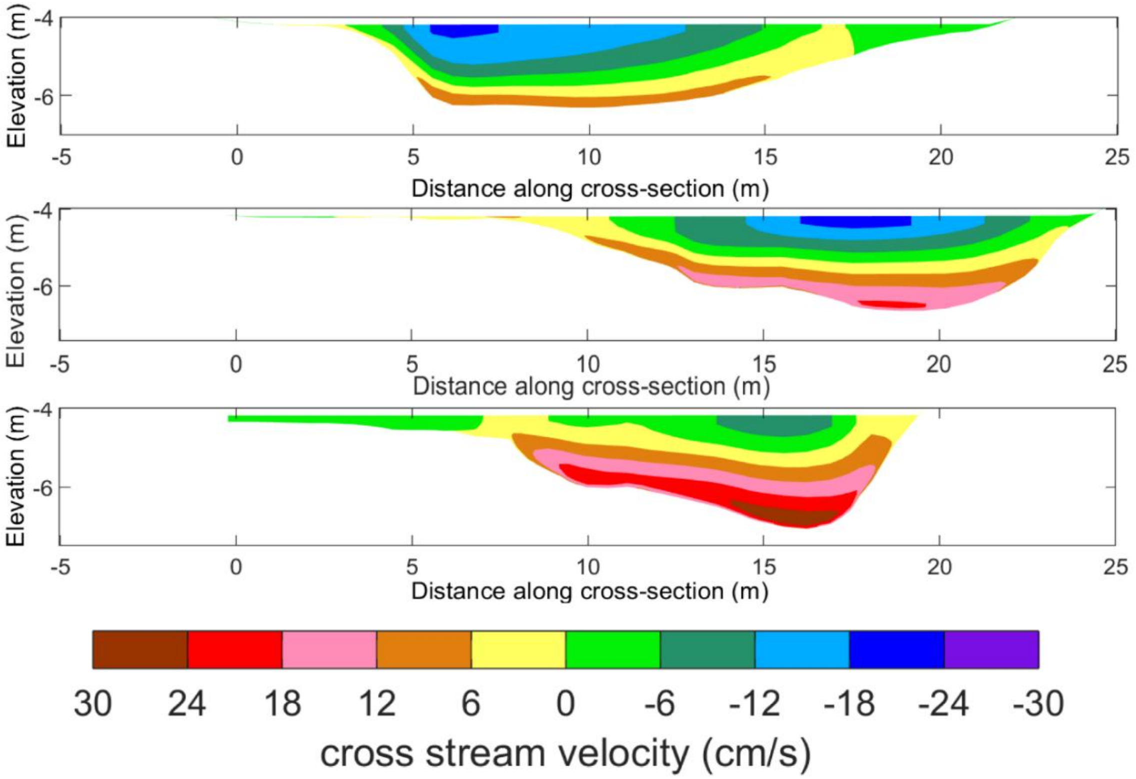

Fig. S8c. Cross-stream component of velocity for three locations through Howard Bend after a 5-day duration in Run 5. Locations shown in *SI Appendix*, Fig. S9: top plot is at section B, middle plot lies between sections C and D and bottom plot is at section E. Positive sign is towards the left bank.

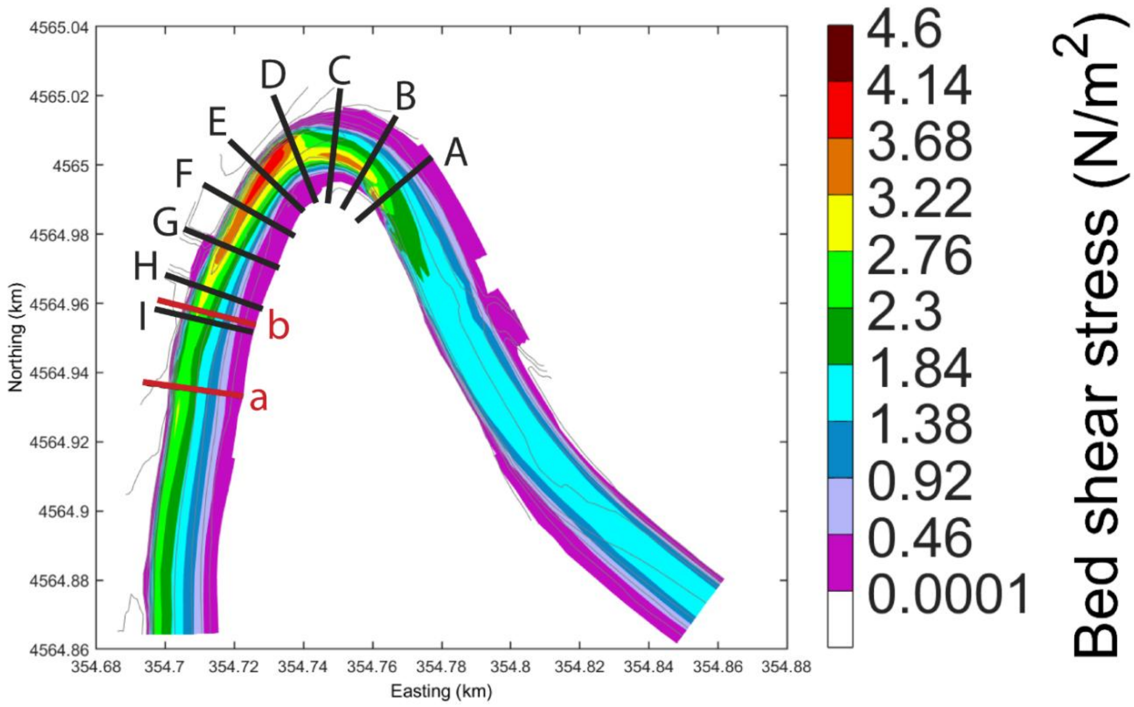

Fig. S9. The locations of sections A-I and a-b in Run 5 are shown on the boundary shear stress. The flow is from right to left.

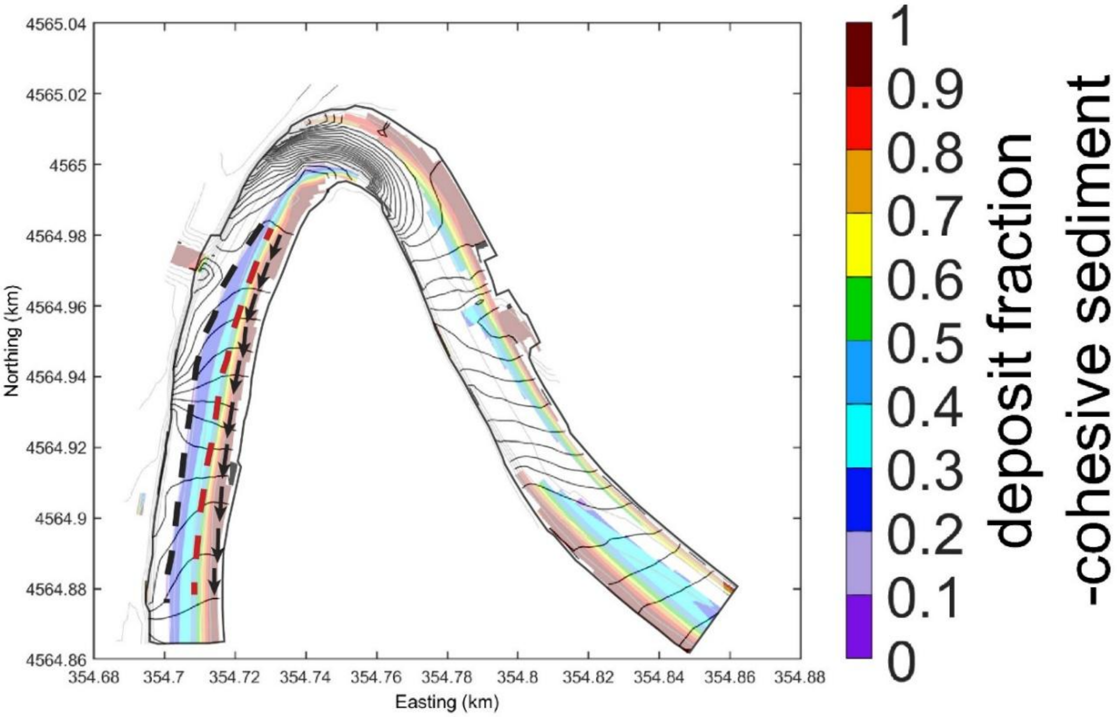

Fig. S10. Overlay of water surface topography and cohesive sediment fraction (Fig. 2D) on the bed topography at the end of Run 5. Dashed black and red lines delineate the zone where 0.1 mm concentration is above input values due to entrainment from pool erosion. Black arrows show the flow following the local pressure gradient along the shallow water whereby relatively little sand is therefore delivered and mud concentration in the deposit area highest. Faint grey lines are 48.6 cm topographic contours. The flow is from right to left.

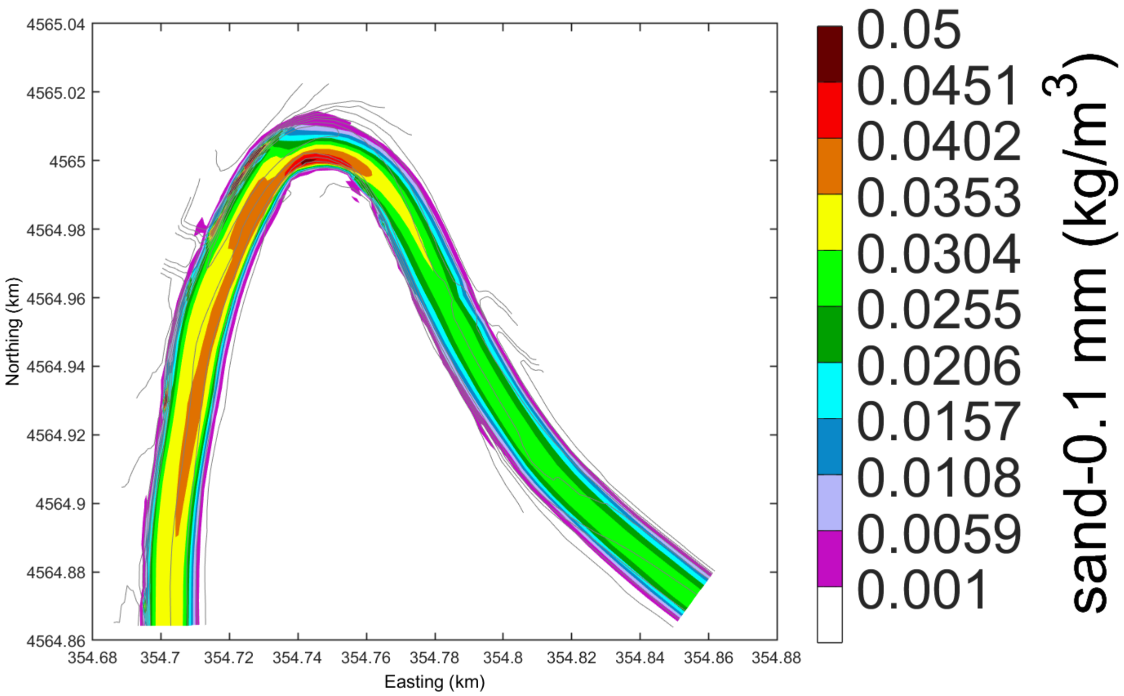

Fig. S11. Basal 0.1 mm concentration through Howard's Bend in Run 5. Scour in the exposed original bed material in the pool and secondary circulation significantly increased the sand concentration onto the lateral accretion deposits. Input basal concentration was 0.021 kg/m^3. The flow is from right to left.

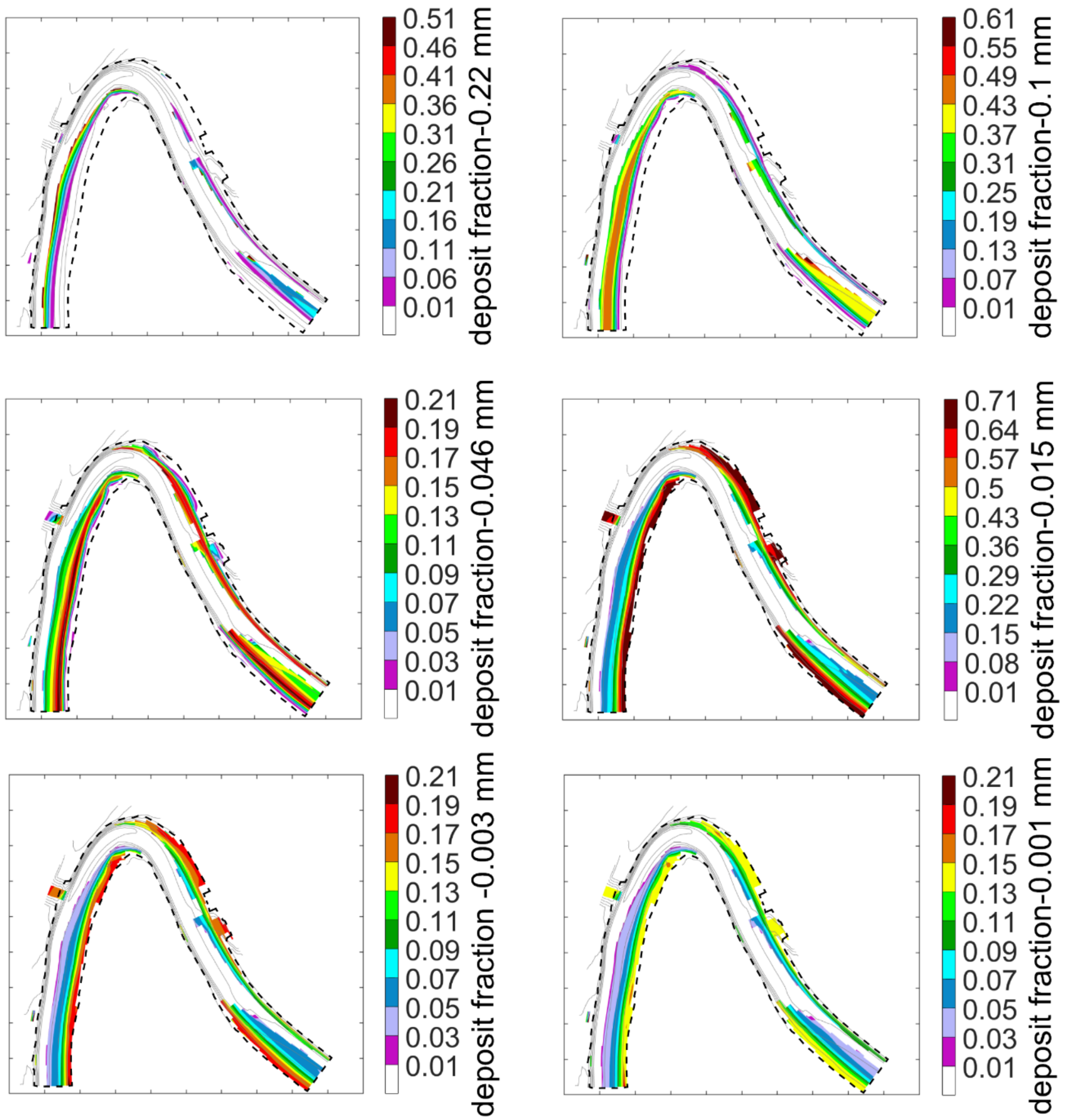

Fig. S12. Fraction of the total sediment deposited during the five-day in Run 5 in each of the six size classes. Black dash line represents channel boundary. Grid scale spacing marking the outline of each plot is 20 m. The flow is from right to left.

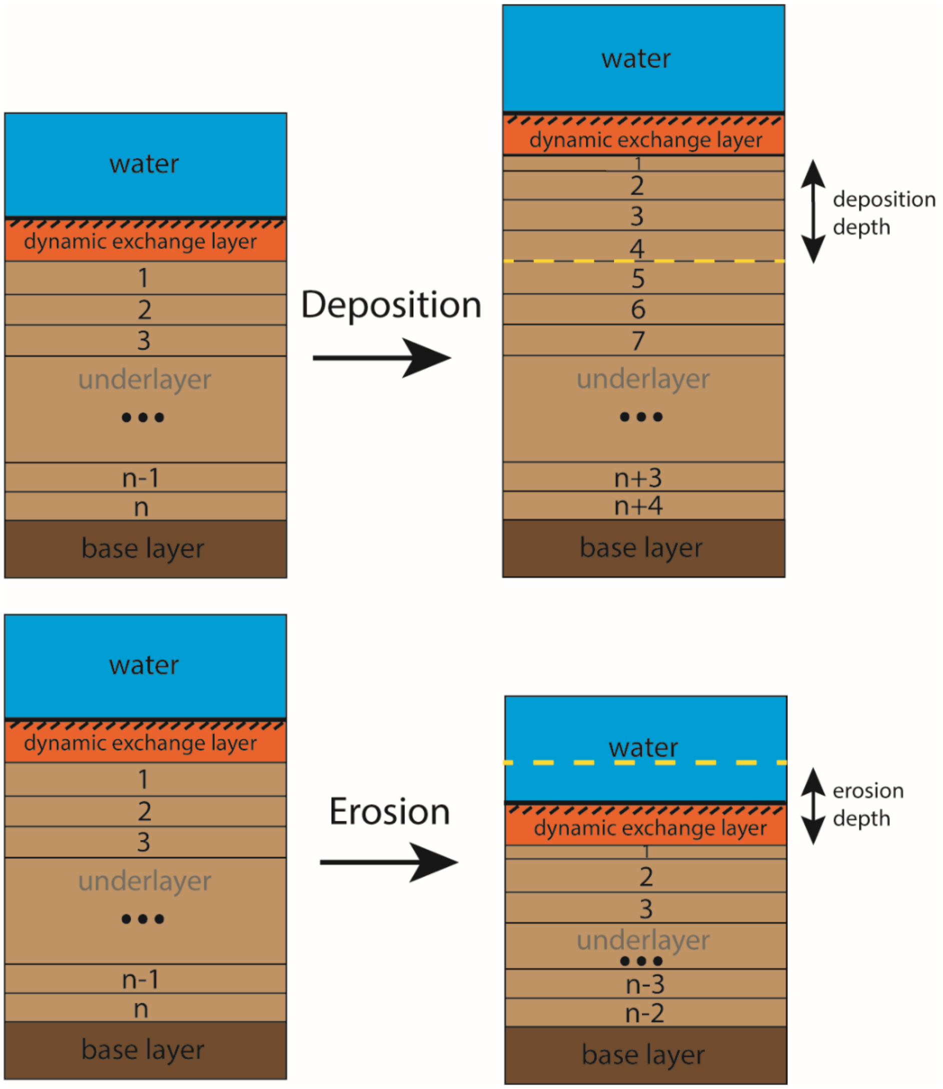

Fig. S13. In Delft3D the dynamics of sediment erosion and deposition acts through a "transport layer", here labeled as the "dynamic exchange layer" because that is its actual function. The user specifies a unit layer in which all accounting takes place, and the surface layer is the dynamic exchange layer (DEL). We used 1 cm, finding the finer layering essentially to document vertical stratigraphy through deposits. Model calculations are performed on the DEL leading to changes in the surface bed fraction of a given particle size class (p_i) as erosion or deposition occurs. During deposition the added sediment is fully mixed with that layer, and once the arriving sediment deposited reaches or exceeds l cm, a new underlayer is created. In the upper illustration, progressive deposition leads to stacks of individually generated layers, hence coarsening or fining can be resolved at the 1 cm level. During slowly varying conditions (we used steady flows in our model) the p_i value and the size fraction of the developing deposit will tend to be equivalent. Consolidation and subsequent bulk density change with burial is not modeled. During erosion, the sediment entrained per unit calculation time equals or exceeds the DEL thickness, and the layer advances 1 cm downward and takes on the material properties of that under layer. In these circumstances, as erosion proceeds the DEL can coarsen due to differential entrainment. We noted at the transition from net erosion to net deposition, that in the depositional area initially the bed deposited mostly sand but as the bed coarsened, the p_i value of the mud declined leading to $D>E$. This points to p_i being dynamic feedback that for constant supply and discharge will tend to produce constant deposition rates for each size class, as noted in Fig. 3.

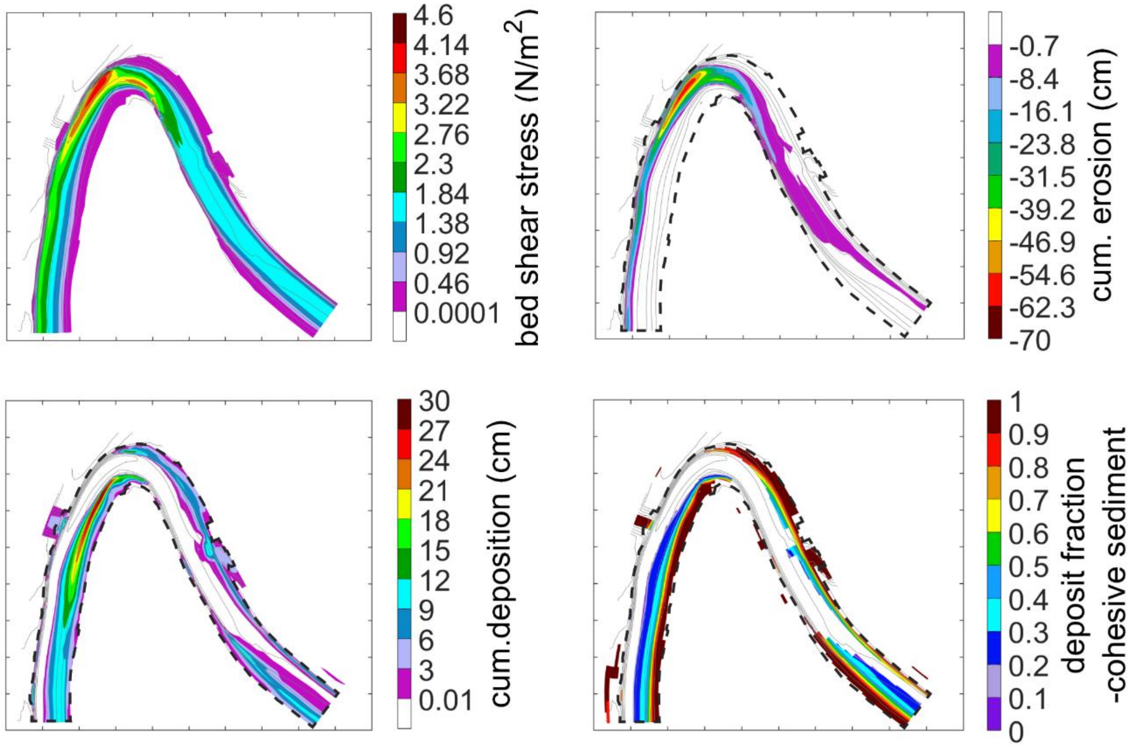

Fig. S14. The boundary shear stress field, cumulative erosion, deposition and fraction of total deposition in cohesive sediment in Run 8 (with lower cohesive sediment settling velocities). Note the similarity of maps with that reported in Fig. 2. Black dash line represents channel boundary. Grid scale spacing marking the outline of each plot is 20 m. The flow is from right to left.

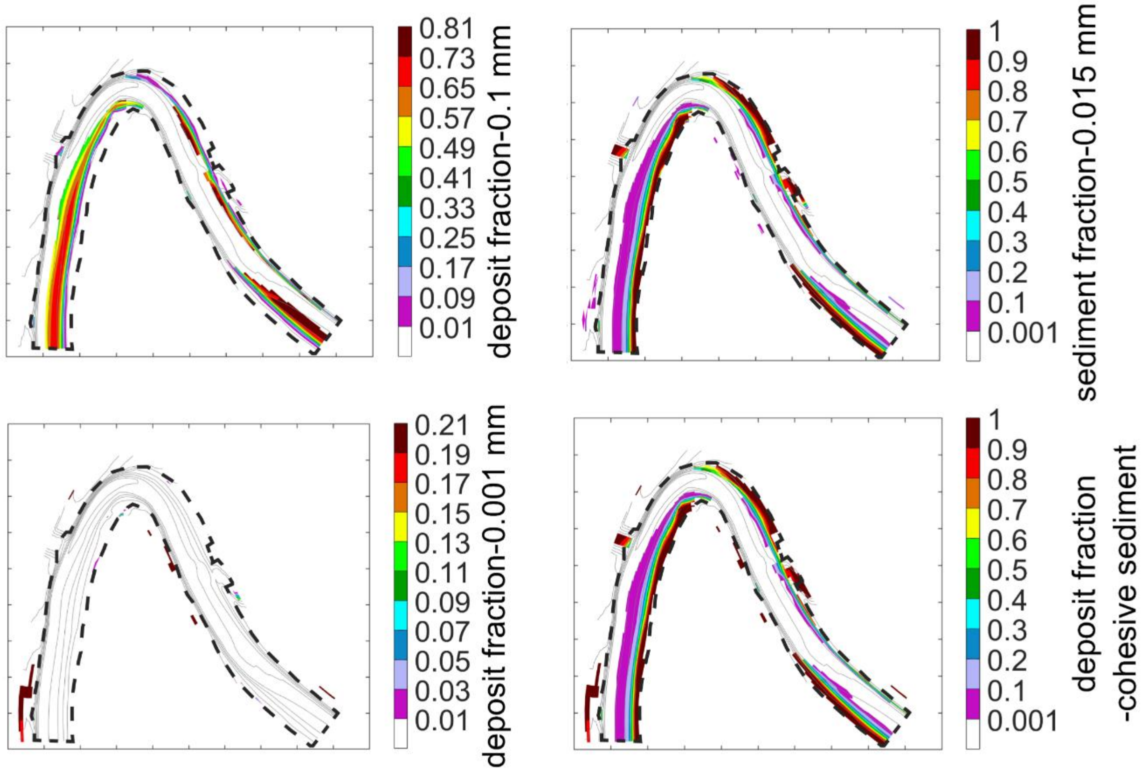

Fig. S15. Fraction of the total sediment deposited during the five-day in Run 9 in 0.1 mm, 0.015 mm, 0.001 mm and cohesive sediment. Black dash line represents channel boundary. Grid scale spacing marking the outline of each plot is 20 m. The flow is from right to left.

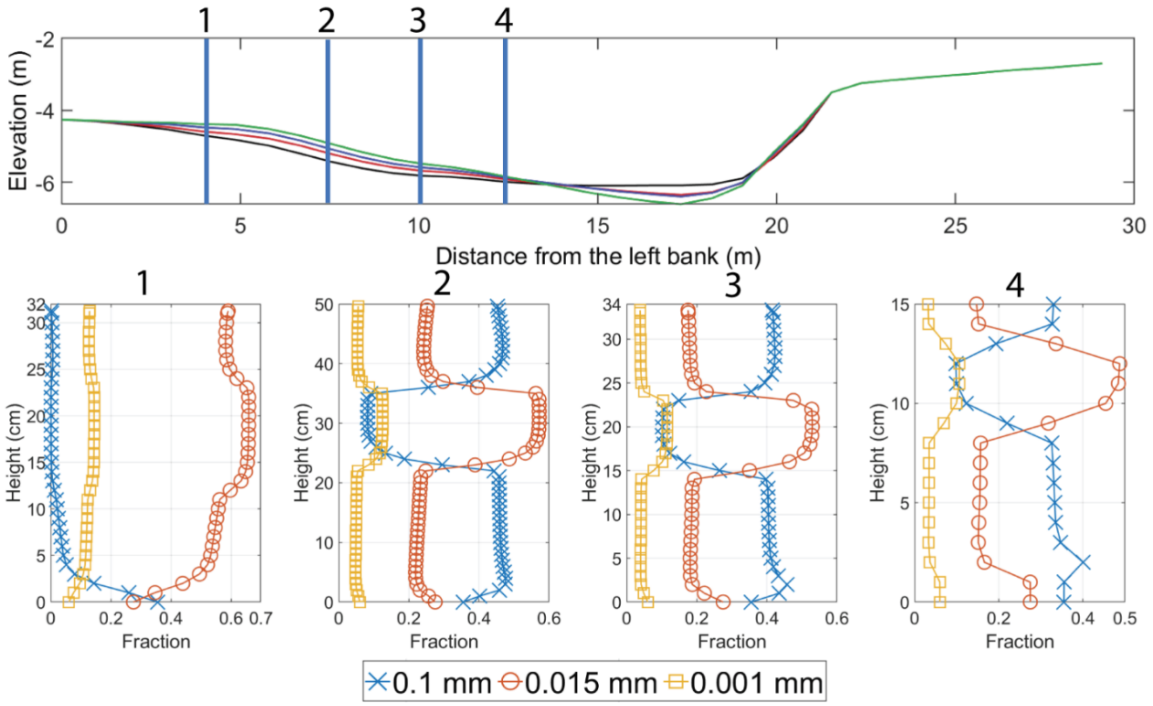

Fig. S16. At cross section b (location in *SI Appendix*, Fig. S9), the bed fractions of layers at sites 1-4 from Run 5 to Run 7. The surface topography at the end of runs are represented by red, blue and green lines.

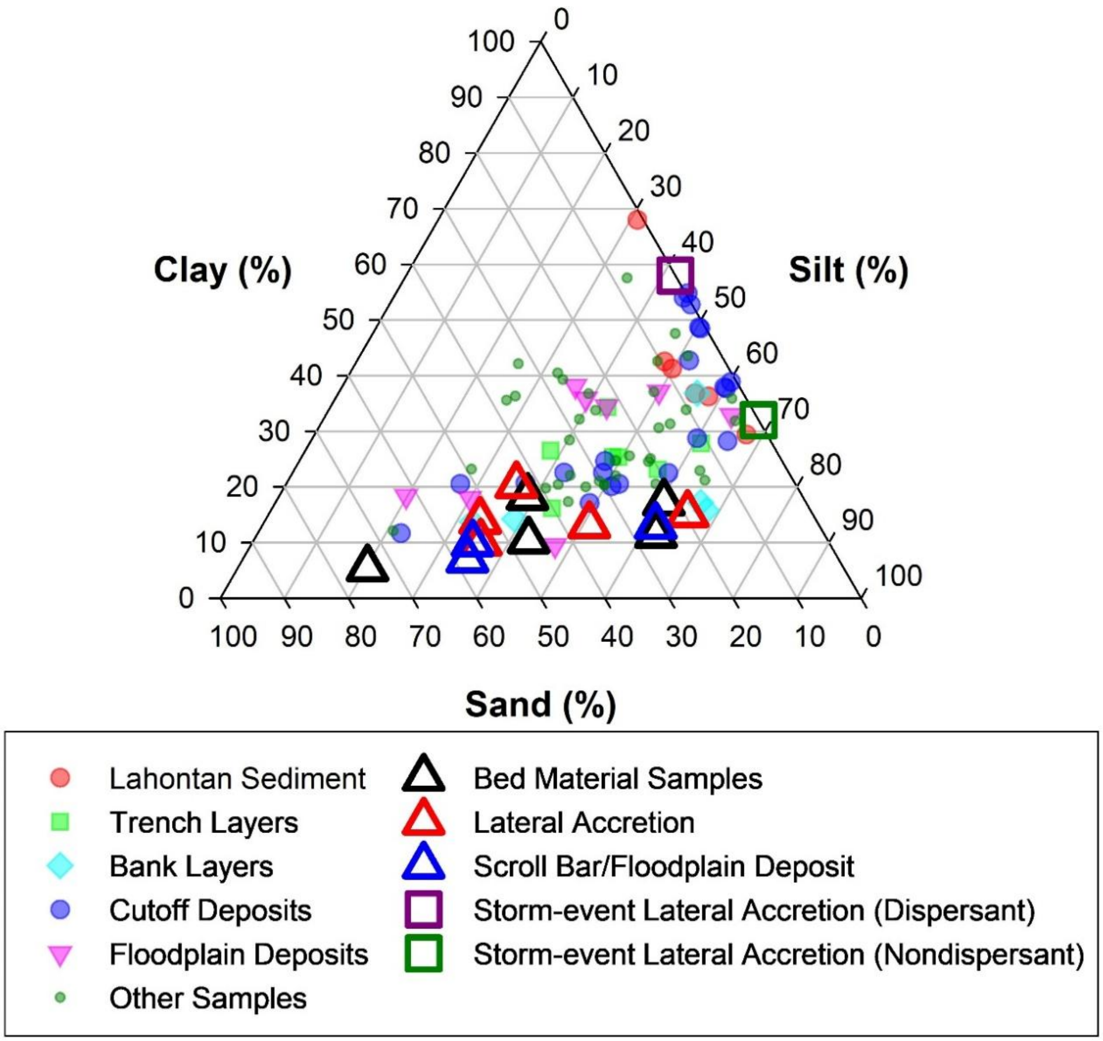

Fig. S17. Comparison of data reported by Matsubara et al. (1) with measurements collected in 2017 specifically of bed material and lateral accretion and scroll bar deposits.

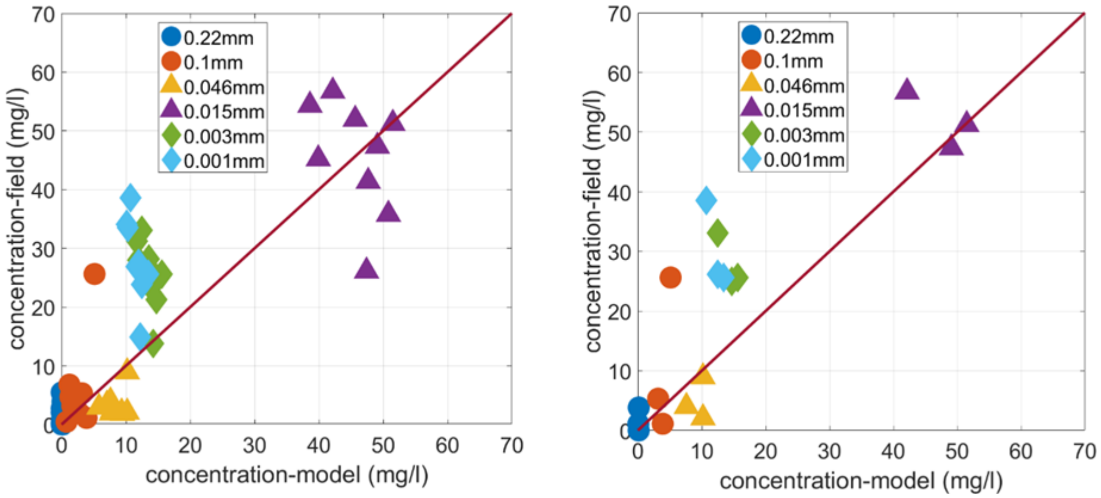

Fig. S18. The left side of the figure illustrates a comparison between measured suspended sediment concentrations and the corresponding concentrations at the same locations in Luca section using Delft3D with the erosion parameter M set at 1.27×10^{-4} kg/m^2/s. On the right side of the figure, only the measured and predicted near-bed concentrations are shown.

SI References

1. Y. Matsubara *et al.*, River meandering on Earth and Mars: A comparative study of Aeolis Dorsa meanders, Mars and possible terrestrial analogs of the Usuktuk River, AK, and the Quinn River, NV. *Geomorphology*. 240, 102-120 (2015).
